# Distinct Immunomodulatory Strategies Guide Mesenchymal Stromal/Stem Cell‐Mediated Bone Regeneration

**DOI:** 10.1002/advs.202518026

**Published:** 2026-03-05

**Authors:** Salwa Suliman, Carla Alvarez Rivas, Aashish Srivastava, Nora Marek, Kamal Mustafa, Alpdogan Kantarci

**Affiliations:** ^1^ Department of Clinical Dentistry Faculty of Medicine Centre For Translational Oral Research (TOR) University of Bergen Bergen Norway; ^2^ The ADA Forsyth Institute Cambridge Massachusetts USA; ^3^ Oral Biology Department School of Dental Medicine University at Buffalo Buffalo New York USA; ^4^ Genome Core Facility Department of Clinical Science University of Bergen Bergen Norway; ^5^ Department of Developmental and Surgical Sciences School of Dentistry University of Minnesota Minneapolis Minnesota USA

**Keywords:** bi‐calcium phosphate, inflammation, MSC priming, regulatory T cells, resolvin E1

## Abstract

The bone regenerative potential of bone marrow‐derived mesenchymal stromal/stem cells (MSC) is critically shaped by their interaction with the host immune system. Here, we compare two MSC priming strategies: coculture with regulatory T cell (Treg) or exposure to the proresolving mediator Resolvin E1 (RvE1) to enhance ectopic osteogenesis in mice. Both approaches enhance MSC‐mediated bone formation through distinct local and systemic immunomodulatory mechanisms. Treg priming promotes a regulated anti‐inflammatory environment characterized by reduced IL‐6 protein levels (3.6‐fold), sustained IL‐13 elevation up to 10 weeks (1.5‐fold compared with RvE1), early suppression of cytotoxic CD8^+^ T cells and IFN‐γ production by CD4^+^ T cells in lymph nodes, a >25% reduction in fibrous capsule formation, and enhanced B cell activity, collectively supporting ossification. In contrast, RvE1 priming promotes early TNF‐α and late granulocyte‐macrophage colony‐stimulating factor (GM‐CSF) production (1.2‐fold compared with Treg) within the scaffold, facilitating osteoblast maturation. Transcriptomic profiling revealed distinct osteogenic signatures, with amphiregulin uniquely upregulated in the Treg group (>7‐fold). Using a Treg depletion model, we further demonstrate that RvE1 retains partial immunomodulatory capacity and supports matrix organization. Together, these findings underscore the versatility of immune modulation in directing MSC fate and highlight the potential of tailoring immunoregulatory strategies to optimize bone regeneration.

## Graphical abstract

Bone regeneration by mesenchymal stem cells is strongly influenced by immune signals. This study shows that priming stem cells with regulatory immune cells or an inflammation‐resolving lipid molecule enhances bone formation through distinct immune pathways. The findings highlight how tailored immune modulation can direct stem cell fate and improve regenerative outcomes.

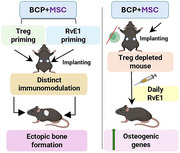

## 1 Introduction

Clinically challenging bone defects represent a growing healthcare problem, particularly in aging and medically compromised populations [1]. Bone marrow‐derived mesenchymal stromal/stem cells (MSC) are adult multipotent progenitor cells capable of differentiating into multilineage skeletal tissues. They have been extensively studied for cell‐based bone engineering by combining them with biomaterials, bioactive molecules, and various priming strategies to reconstruct clinically challenging bone defects [2]. Promising outcomes using in vitro‐expanded MSC have been described in preclinical and small‐scale clinical trials for bone regeneration [3]. However, a critical factor influencing the success of transplanted MSC is their complex, synchronized interactions with the recipient's immune system, which can lead to suboptimal results. Despite this, the impact of the immune response on the efficacy of MSC‐based therapies remains largely unexplored.

The immune system plays an instrumental role in regulating bone regeneration [4]. T lymphocytes, key components of the adaptive immune system, have become increasingly recognized for their role in bone healing [5]. In vivo studies have revealed that mice lacking functional T cells exhibit severe alterations in bone matrix structure and aberrant fracture healing, due to impaired cartilage mineralization [6]. Additionally, in both a mouse osteotomy model and in human fracture callus, impaired fracture healing correlates significantly with elevated levels of terminally differentiated CD8^+^ T cells in peripheral blood and locally within fracture hematoma [5]. Moreover, in stem cell‐based bone regeneration, CD4^+^ T‐helper 1 and CD8^+^ T cell subsets can inhibit bone formation by inducing apoptosis of implanted MSC [7]. Thus, while T cells are essential for proper bone healing, specific subsets may have detrimental effects, highlighting the delicate balance between inflammation and resolution in the context of bone regeneration.

Regulatory T cells (Treg) are a specialized subset of CD4^+^ T cells with a distinctive role in maintaining immune homeostasis and modulating immune responses [8]. They are characterized by the expression of the transcription factor forkhead box protein P3 (Foxp3), which enables their canonical immune regulatory capabilities to suppress exacerbated immune responses, maintain self‐tolerance, and a more recently participate in tissue regeneration [9, 10]. Among a growing number of tissue types, Treg have demonstrated potential in enhancing bone regeneration with their pro‐osteogenic role well established in bone‐destructive pathologies, such as osteoarthritis, osteoporosis, and periodontitis [5, 11]. In addition, systemic MSC infusions have been shown to upregulate endogenous Treg alongside reduced levels of proinflammatory cytokines, resulting in improved bone regeneration [12]. Given these promising outcomes, various strategies have been proposed to improve cell‐based bone engineering by mitigating pro‐inflammatory responses, including systemic Treg infusion and local administration of anti‐inflammatory drugs [12]. Unlike anti‐inflammatory drugs, Treg play a dual role in promoting tissue regeneration: they regulate effector immune cells and also exert direct effects on tissue progenitors, as recently demonstrated in muscle (satellite) cells [13].

Recognition of the importance of well‐regulated proinflammatory and anti‐inflammatory phases in bone regeneration has led to the development of modulatory strategies aimed at leveraging the immune system to promote bone repair [14]. Priming of MSC with cytokines, growth factors, pharmacological agents, various biomaterials, or specific culture conditions prior to implantation has shown potential to improve their therapeutic efficacy [15]. For example, MSC have been cocultured with innate immune cells (monocyte‐derived macrophages) and nonimmune cells (endothelial cells) to enhance their osteogenic potential [16, 17]; however, these approaches have produced contradictory results [18]. Specialized proresolving lipid mediators (SPMs) have recently emerged as promising priming candidate due to their potent ability to resolve inflammation [19].

These bioactive lipids, generated through the enzymatic metabolism of polyunsaturated fatty acids, act as key mediators that actively resolve inflammation by interacting with specific receptors on immune and stromal cell types [20]. Among SPMs, Resolvin E1 (RvE1) is one of the most studied in the context of inflammatory bone loss and has shown promising effects when used locally and systemically to promote bone repair and regeneration [21, 22]. However, its impact on the osteogenic potential of MSC in vivo remains poorly defined. Previous studies have either examined the immunomodulatory effects of RvE1 on MSC in vitro without assessing osteogenesis [19], or have focused on periodontal ligament stem cells [23] and not bone marrow‐derived MSC. Furthermore, earlier in vivo work with SPMs in bone regeneration delivered these mediators directly to bone defects to act on endogenous progenitor cells [21]. Additionally, our prior work demonstrated that RvE1 protects Treg in periodontal tissue by promoting a shift of Th17 cells toward a regulatory phenotype, creating conditions favorable for Treg function [24]. Therefore, RvE1 enhances Treg function and may further improve Treg‐MSC interactions in the context of bone regeneration, an area that remains unexplored.

**FIGURE 1:**
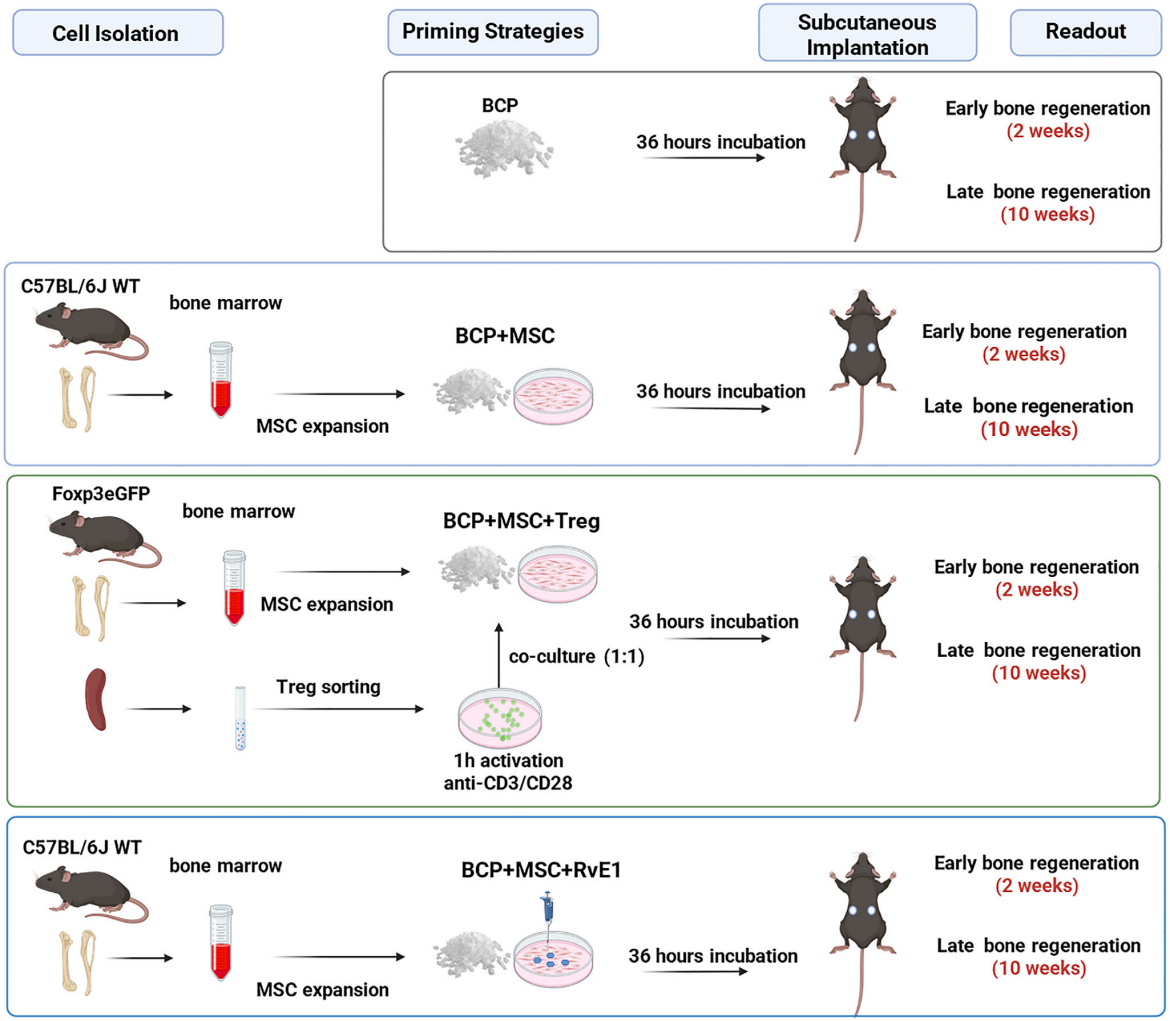
Schematic overview of the experimental design and groups. Bone marrow cells were isolated from C57BL/6J wild type or Foxp3^eGFP^ mice and expanded to obtain MSC. Treg were isolated from Foxp3^eGFP^ mice by cell sorting. BCP scaffolds were prepared using four different strategies prior to implantation: BCP alone (BCP); BCP cultured with MSC for 36 h (BCP+MSC); BCP cultured with MSC and Treg at a 1:1 ratio (BCP+MSC+Treg); BCP cultured with MSC that had been primed with RvE1 (BCP+MSC+RvE1). All constructs were incubated for 36 h before implantation into recipient mice. Bone regeneration was assessed at early 2 (weeks) and late (10 weeks) time points. Created with Biorender.com; used under license.

To address this gap, we investigated MSC priming strategies; either through coculturing with Treg or RvE1 prior to in vivo implantation to foster immune‐mediated bone regeneration (Figure 1). We also evaluated the significance of endogenous Treg in MSC‐based bone regeneration and examined the effect of RvE1 treatment in a Treg‐deficient mouse model to assess its potential to rescue Treg deficiency in the context of bone regeneration.

## 2 Results

### 2.1 MSC Priming Strategies Induce Divergent Immune Cell Recruitment and IFN‐γ Production after 2 Weeks

After 2 weeks and 10 weeks, total cellularity was determined by manual cell counts from harvested lymph nodes and spleens, with 4–6 animals analyzed per group per time point. At 2 weeks, lymph nodes from all experimental groups recruited a greater number of immune cells compared to baseline animals. Notably, the BCP+MSC+RvE1 group exhibited the highest lymph node cellularity at 2 weeks (*p* = 0.016) compared to baseline animals, whereas the BCP+MSC+Treg showed the highest lymph node cellularity at 10 weeks (nonsignificant). After 10 weeks, all groups exhibited a general reduction in total lymph node cellularity, with no significant difference between groups. In the spleens, BCP+MSC showed a significant reduction in cellularity at 2 weeks compared to the highest values observed in the BCP+MSC+RvE1 (*p* = 0.025) and BCP+MSC+Treg (*p* = 0.048) groups, with no significant differences at 10 weeks (Figure S2a).

**FIGURE 2:**
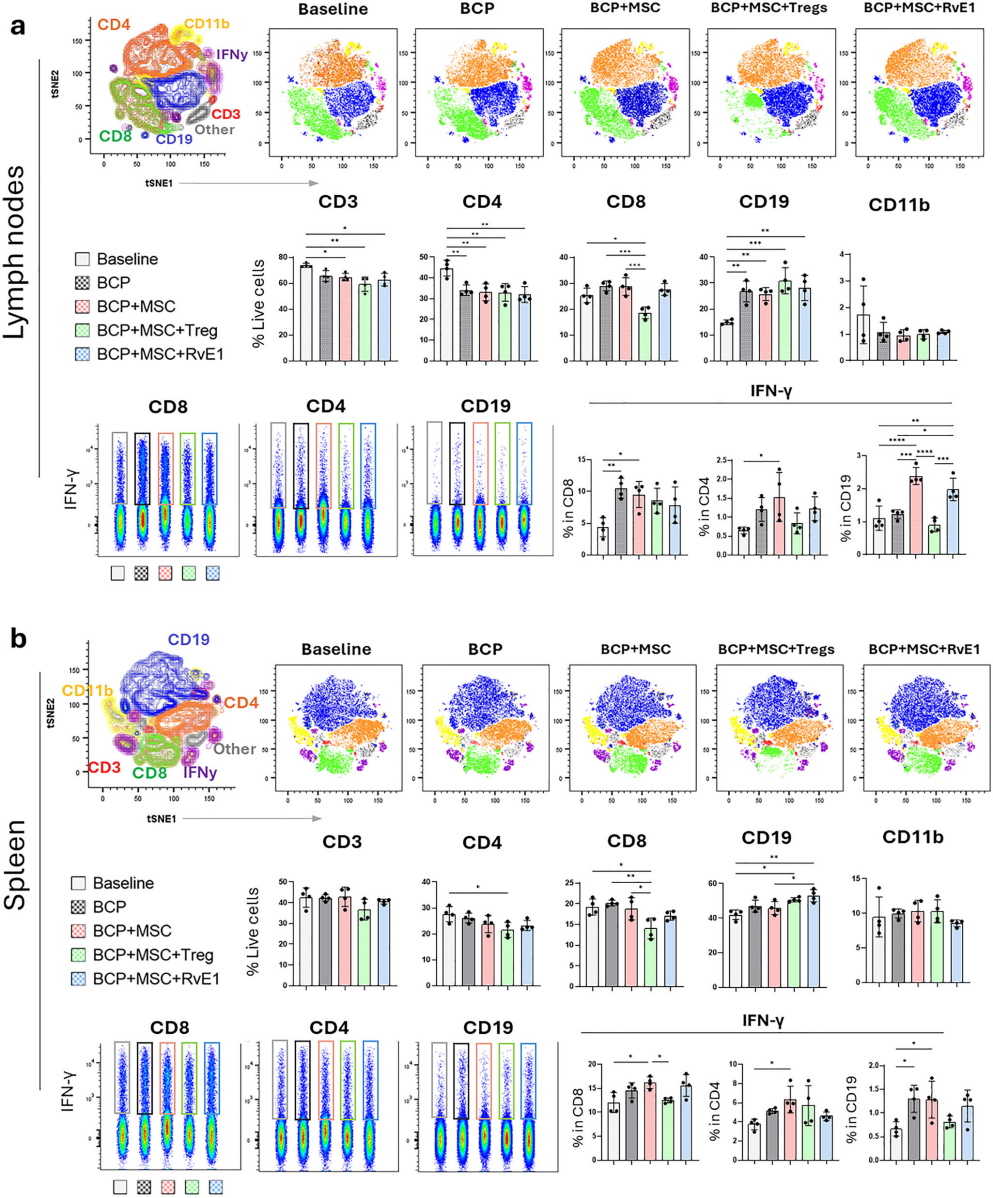
MSC priming strategies results in divergent immune cell recruitment and varied IFN‐γ production after 2 weeks. Dimensional reduction using *t*‐SNE illustrates the distribution of immune cell populations, demonstrating distinct clustering patterns across groups. “Baseline” refers to untreated, construct‐free animals. Absolute quantification of each immune cell subset, along with absolute IFN‐γ production, is shown for (a) lymph nodes and (b) spleen. Data are presented as mean +/− SEM (*n* = 4 animals per group). Statistical significance was determined by one‐way ANOVA with post hoc tests. *p < 0.05, **p < 0.01, ***p < 0.001, ****p < 0.0001.

Absolute quantification of distinct cell populations in lymph nodes and spleen at 2 weeks was performed using flow cytometry, analyzing 4 animals per group. Populations included CD3^+^CD4^+^ helper T cells, CD3^+^CD8^+^ cytotoxic T cells, CD3^+^CD19^+^ B cells, and CD3^–^CD11b^+^ myeloid cells/monocytes. In the lymph nodes at 2 weeks, the baseline animals group had the highest number of CD3^+^ T cells compared to BCP+MSC (*p* = 0.044), BCP+MSC+Treg (*p* = 0.001), BCP+MSC+RvE1 (*p* = 0.011). CD4^+^ T cells were also significantly higher in baseline animals compared to BCP (*p* = 0.008), BCP+MSC (*p* = 0.004), BCP+MSC+Treg (*p* = 0.004), and BCP+MSC+RvE1 (*p* = 0.001). CD11b^+^ cells were reduced in all groups compared to baseline animals, although the difference was not significant. In contrast, the BCP+MSC+Treg group showed the lowest CD8^+^ cytotoxic T‐cell counts compared to baseline animals (*p* = 0.02), BCP (*p* = 0.0003), and BCP+MSC (*p* = 0.0004) (Figure 2a). Additionally, this group also displayed an elevated CD4^+^ T/CD8^+^ T cell ratio comparable to baseline animals. Furthermore, BCP+MSC+Treg showed the highest CD19^+^ B‐cell counts (*p* = 0.0002) when compared to baseline animals.

IFN‐γ production in lymph node immune subsets revealed cell‐type‐specific variation at 2 weeks. CD4^+^ T cells in the BCP+MSC (*p* = 0.031) and CD8^+^ T cells in the BCP group (*p* = 0.006) produced significantly higher IFN‐γ compared to baseline animals. The BCP+MSC+Treg displayed the lowest IFN‐γ production trends from CD4^+^ and CD19^+^ cells with significantly lower CD19^+^ IFN‐γ expression compared to BCP+MSC (*p* = 0.0001) and BCP+MSC+RvE1 (*p* = 0.0005). CD19^+^ B cells in BCP+MSC and BCP+MSC+RvE1 showed the highest IFN‐γ production, with BCP+MSC significantly higher than baseline animals (*p* = 0.0001) and BCP (*p* = 0.0002). CD19^+^ B cells in BCP+MSC+RvE1 exhibited significantly higher IFN‐γ production compared to BCP (*p* = 0.011), baseline animals (*p* = 0.0034), and BCP+MSC+Treg (*p* = 0.0005) (Figure 2a).

In the splenic response at 2 weeks, the BCP+MSC+Treg group exhibited the lowest CD4^+^ T‐cell counts compared to baseline animals (*p* = 0.037) and the lowest CD8^+^ T‐cell counts compared to baseline animals (*p* = 0.014), BCP (*p* = 0.004), and BCP+MSC (*p* = 0.037). CD19^+^ B cells showed a distinct pattern, with significantly higher counts in the BCP+MSC+RvE1 group compared to baseline animals (*p* = 0.001) and BCP+MSC (*p* = 0.04). CD19^+^ B cells were also significantly elevated in the BCP+MSC+Treg compared to baseline animals (*p* = 0.01). In line with the lymph nodes, CD11b^+^ cell counts in the spleen showed no significant differences across groups at 2 weeks (Figure 2b).

Spleens IFN‐γ production at 2 weeks revealed notable variation. IFN‐γ production by CD8^+^ cytotoxic T cells was significantly decreased in both BCP+MSC+Treg (*p* = 0.045) and baseline animals (*p* = 0.02) compared to BCP+MSC. The BCP+MSC exhibited markedly elevated IFN‐γ production in CD8^+^ cytotoxic T cells (*p* = 0.02), CD4^+^ helper T (*p* = 0.046), and CD19^+^ B cells (*p* = 0.044) compared to baseline animals (Figure 2b).

### 2.2 Distinct Transcriptional Signatures of Skeletal Regeneration and Immunity Emerge From MSC Priming via Treg Coculture or RvE1

For each group, bulk RNA sequencing was conducted on four biological replicates, each from an individual animal. The genes expressed reflect contributions from the entire construct, including both seeded cells and infiltrating host cells. All samples were included after undergoing quality control. A total of 1703 genes were significantly upregulated (*p*‐value < 0.05 with log2FC of +1 or higher) in BCP+MSC+RvE1, 1107 genes in BCP+MSC+Treg and only 651 genes in BCP+MSC to the BCP group. Conversely, BCP+MSC expressed 421 downregulated genes, BCP+MSC+RvE1 expressed 1062 downregulated genes, and BCP+MSC+Treg expressed 542 downregulated genes compared to BCP (Figure S2b).

**FIGURE 3:**
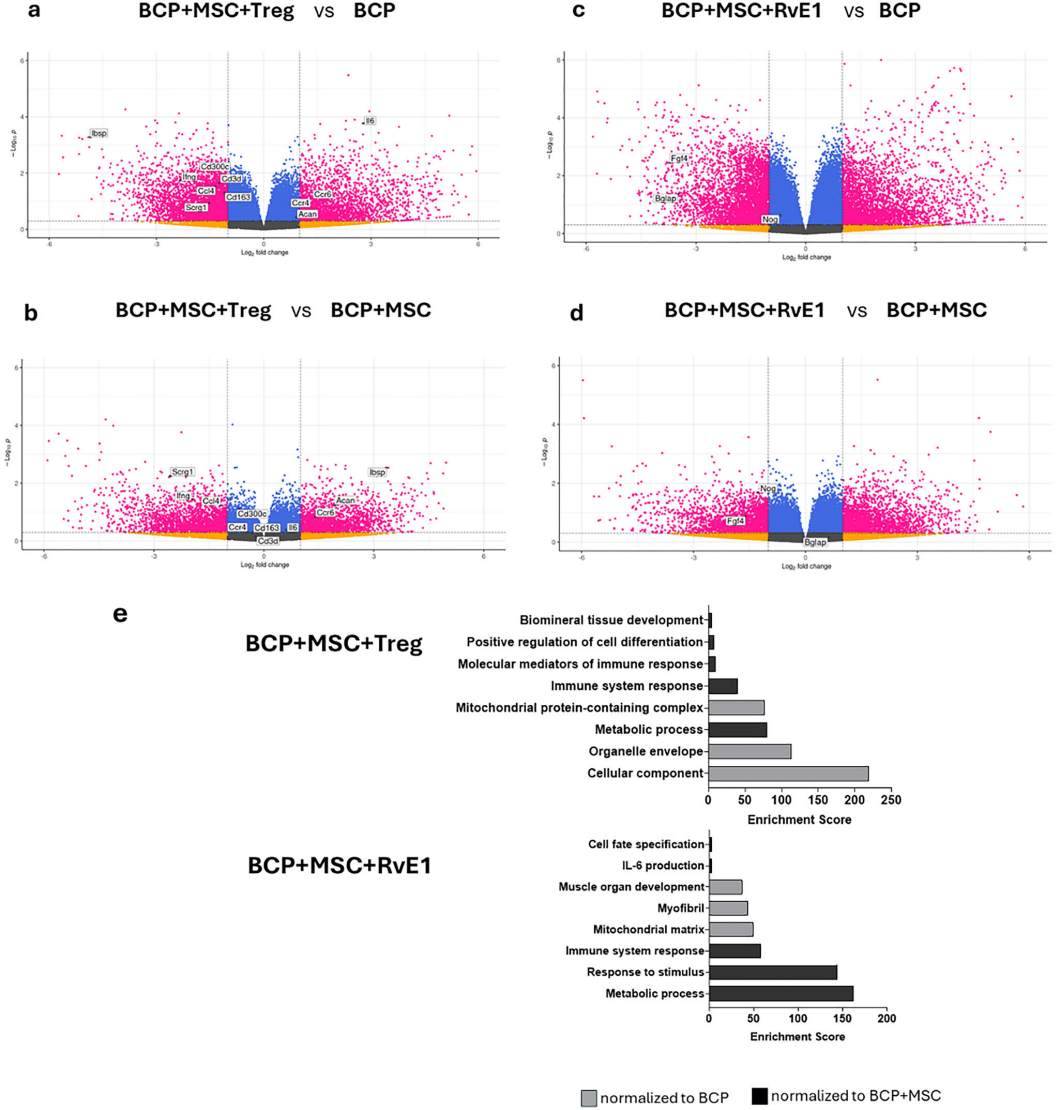
Transcriptional expression profiles from implanted groups after 2 weeks. Volcano plots show differential gene expression for (a) BCP+MSC+Treg vs. BCP, (b) BCP+MSC+Treg vs. BCP+MSC (c) BCP+MSC+RvE1 vs. BCP and (d) BCP+MSC+RvE1 vs. BCP+MSC. (e) Enrichment scores identifying functional modules in BCP+MSC+Treg and BCP+MSC+RvE1 when normalized to BCP (gray bars) or BCP+MSC (black bars). *N* = 4 animals per group.

The gene expression profiles across groups exhibited a range of functional signatures. Notably, BCP+MSC+Treg significantly expressed genes associated with musculoskeletal regeneration and immune function. Osteogenesis‐related genes included *Scrg1* (*p* = 0.006) and *Ibsp* (*p* = 0.0005) compared to BCP+MSC or BCP. Interestingly, BCP+MSC+Treg significantly downregulated the chondrogenic genes *Matn3* (*p* = 0.04) and *Has2* (*p* = 0.003) compared to BCP. Immune‐regulating genes upregulated in BCP+MSC+Treg compared to BCP included *CD163* (*p* = 0.044) and *Ccl4* (*p* = 0.029). Additionally, this group also expressed significantly higher levels of *Ifng* (*p* = 0.017) and downregulation of *Il6* (*p* = 0.0002) compared to BCP+MSC or BCP, respectively. The BCP group significantly expressed *Ccr4* (*p* = 0.03) compared to BCP+MSC and *Ccr6* (*p* = 0.045) compared to BCP+MSC+Treg. Several genes related to T‐ and B‐cell activation were also highly expressed in BCP+MSC+Treg, including *CD3d* (*p* = 0.024) and *CD300c* (*p* = 0.004) compared to BCP (Figure 3a,b).

A comparable trend was observed in BCP+MSC+RvE1, where upregulation of musculoskeletal regeneration genes such as *Fg4* (*p* = 0.004) and *Bglap* (*p* = 0.04) was noted compared to BCP and *Nog* (*p* = 0.02) compared to BCP+MSC (Figure 3c,d).

When compared to BCP and BCP+MSC, enrichment analysis demonstrated an enrichment of genes in BCP+MSC+Treg involved in processes characteristic of osteogenesis or mineralization, immune responses, cell differentiation, and metabolic processes among others (Figure 3e). In BCP+MSC+RvE1, enriched pathways were predominantly related to muscle production and immune system responses (Figure 3e).

Only when BCP+MSC was compared to BCP was upregulation of osteogenesis‐related genes observed, including *Sp7* (*p* = 0.039) and *Ibsp* (*p* = 0.000 000 003). Enrichment scores for BCP+MSC normalized to BCP revealed pathways involved in bone mineralization, actin binding, myofibril, cytoskeletal protein binding among others (Figure S3a,b).

BCP+MSC+Treg and BCP+MSC+RvE1 shared a relatively large number of commonly upregulated and downregulated genes, totaling 707 and 285 genes, respectively. Several immunoregulatory genes were significantly upregulated in BCP+MSC+Treg compared to BCP+MSC+RvE1; including *Ido1* (*p* = 0.044) and *Klrc3* (*p* = 0.024) as well tissue repair and matrix reorganizing genes, such as *Areg* (*p* = 0.033) and *Mmp1b* (*p* = 0.033), respectively. In BCP+MSC+RvE1, *Il6* (*p* = 0.042) and the osteogenic gene *Ibsp* (*p* = 0.011) were upregulated (Figure S3c,d). Interestingly, pathways enriched in BCP+MSC+Treg compared to BCP+MSC+RvE1 were largely associated with immune cell pathways, T cell differentiation and activation, among other pathways (Figure S3d).

### 2.3 MSC Priming via Treg Coculture or RvE1 Formed Bone In Vivo but Exhibited Varied Histomorphology

**FIGURE 4:**
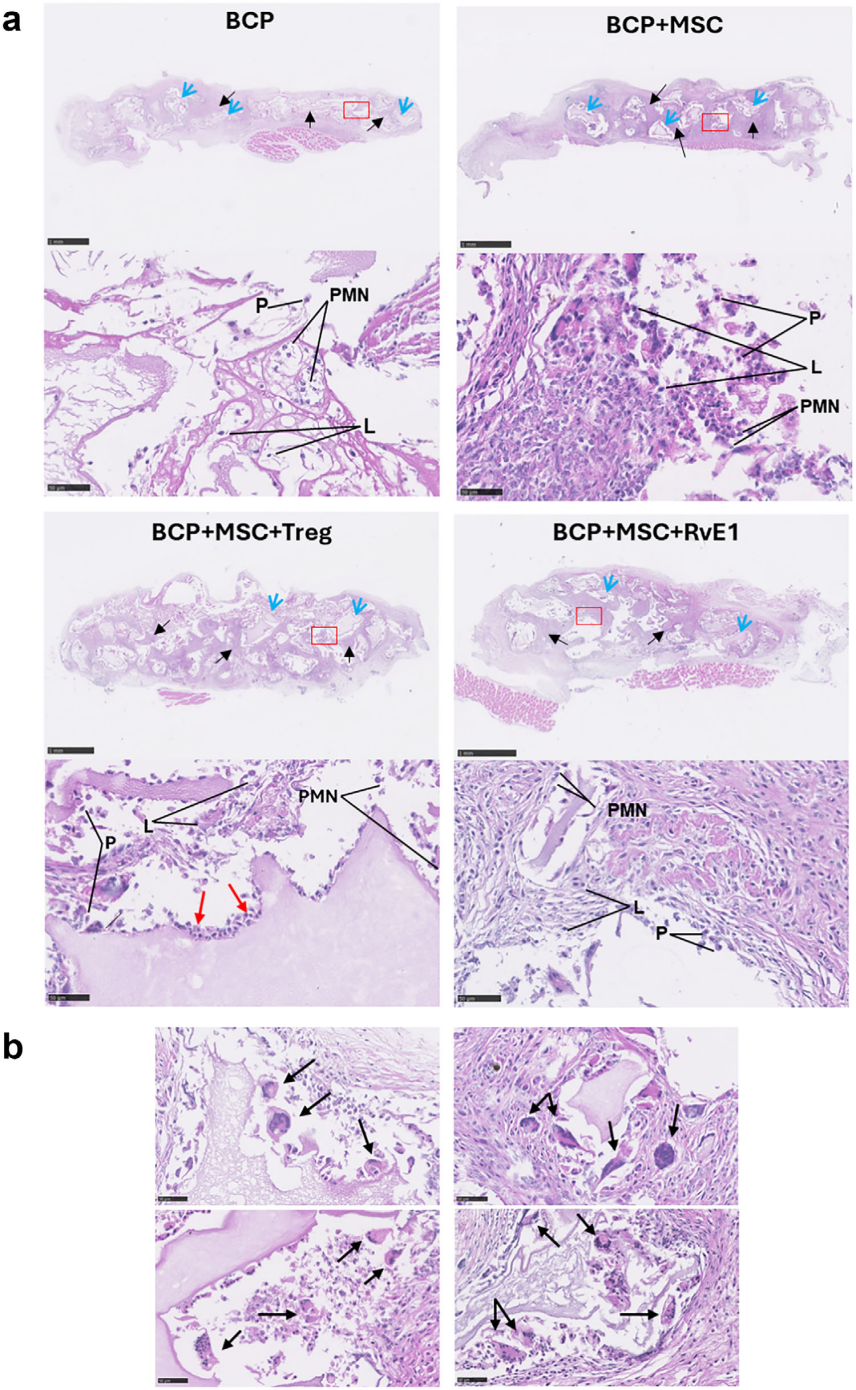
MSC priming via Treg coculture or RvE1 exhibits varied histomorphology. (a) Representative H&E‐stained histological sections after 2 weeks. Low magnification images (scale bar = 1 mm) show BCP granules (blue arrows) and infiltrating connective tissue (black arrows). Higher magnification images (scale bar = 50 µm) display Plasma cells (P), Lymphocytes (L), and polymorphonuclear cells (PMN). (b) Foreign body giant cells across different groups are indicated with black arrows.

Differences were observed in histomorphology among the groups, including variations in the density of connective tissue‐forming fibers, their organization, and overall cellularity (Figure 4a). After 2 weeks, all groups showed ingrowth of connective tissue around the granules. However, the BCP group displayed loose connective tissue surrounding the granules (Figure 4a, black arrows) compared to the other groups at 2 weeks. In contrast, organized and dense connective tissue was observed when BCP was combined with MSC, MSC+Treg, or MSC+RvE1 (Figure 4a, black arrows). Among all groups, BCP+MSC+Treg exhibited cuboidal/polygonal‐shaped cells forming a single layer aligned around the granules (Figure 4a, red arrows). Infiltration of inflammatory cells, including polymorphonuclear cells, lymphocytes, macrophages, and plasma cells was observed across all groups within the connective tissue surrounding the granules (Figure 4a). Notably, foreign body giant cells (FBGC) were evident in all groups starting from 2 weeks (Figure 4b, black arrows). FBGC were observed near and along granule edges, with clear morphological differences between groups and time points.

**FIGURE 5:**
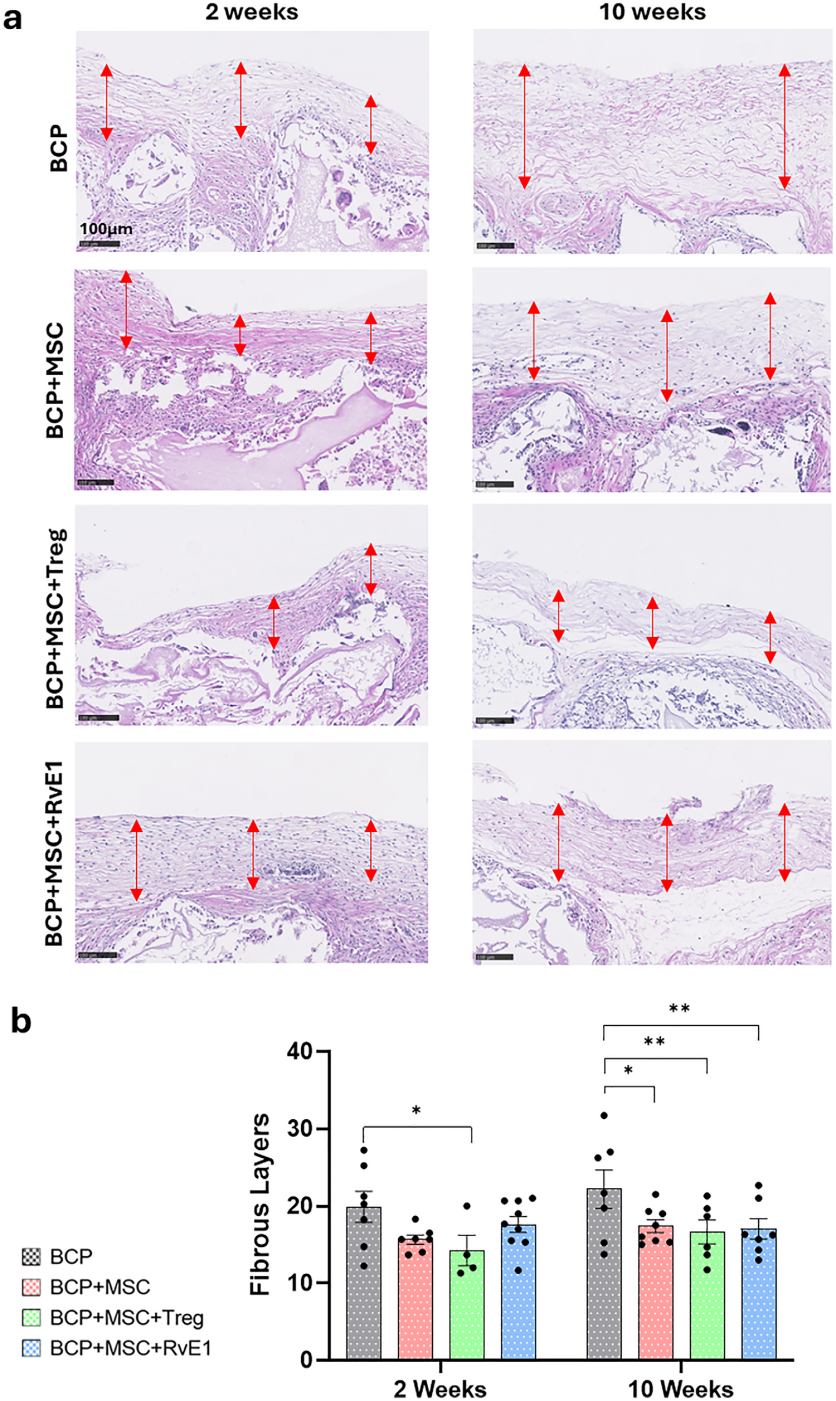
Fibrous capsule analysis. (a) Representative H & E stained sections after 2 and 10 weeks. Red arrows indicate the thickness of the fibrous capsule. (b) Quantification of fibrous capsule layers after 2 and 10 weeks. Data are presented as means +/− SEM (*n* = 5 animals per group). Statistical significance was determined by one‐way ANOVA with post hoc tests. *p < 0.05, **p < 0.01.

At 2 weeks, all fibrous capsules exhibited low cellularity, lacking vascularization and inflammatory infiltrate (Figure 5a). In this early time point, the capsules consisted mainly of organized, whereas at 10 weeks the capsules were denser and composed of unorganized collagen fibers. The fibrous capsule also varied in thickness and organization between groups at both 2 and 10 weeks (Figure 5a, red arrows). A total of 5 animals were analyzed per time point. At 2 weeks, the fibrous capsule thickness ranged from 14.3 + 7.1 to 19.9 + 7.5 layers, with the BCP+MSC+Treg group showing significantly fewest layers (*p* = 0.025) compared to the BCP group, which had the highest number of layers. At 10 weeks, the layers ranged from 16.6 + 6.8 to 22.2 + 8.4, and all groups exhibited significantly fewer layers compared to BCP, with BCP+MSC+Treg (*p* = 0.017) again showing the least (Figure 5b). Between the 2 and 10 weeks, capsule thickness increased in all groups except BCP+MSC+RvE1, where the number of layers remained within the same range (Figure 5b).

**FIGURE 6:**
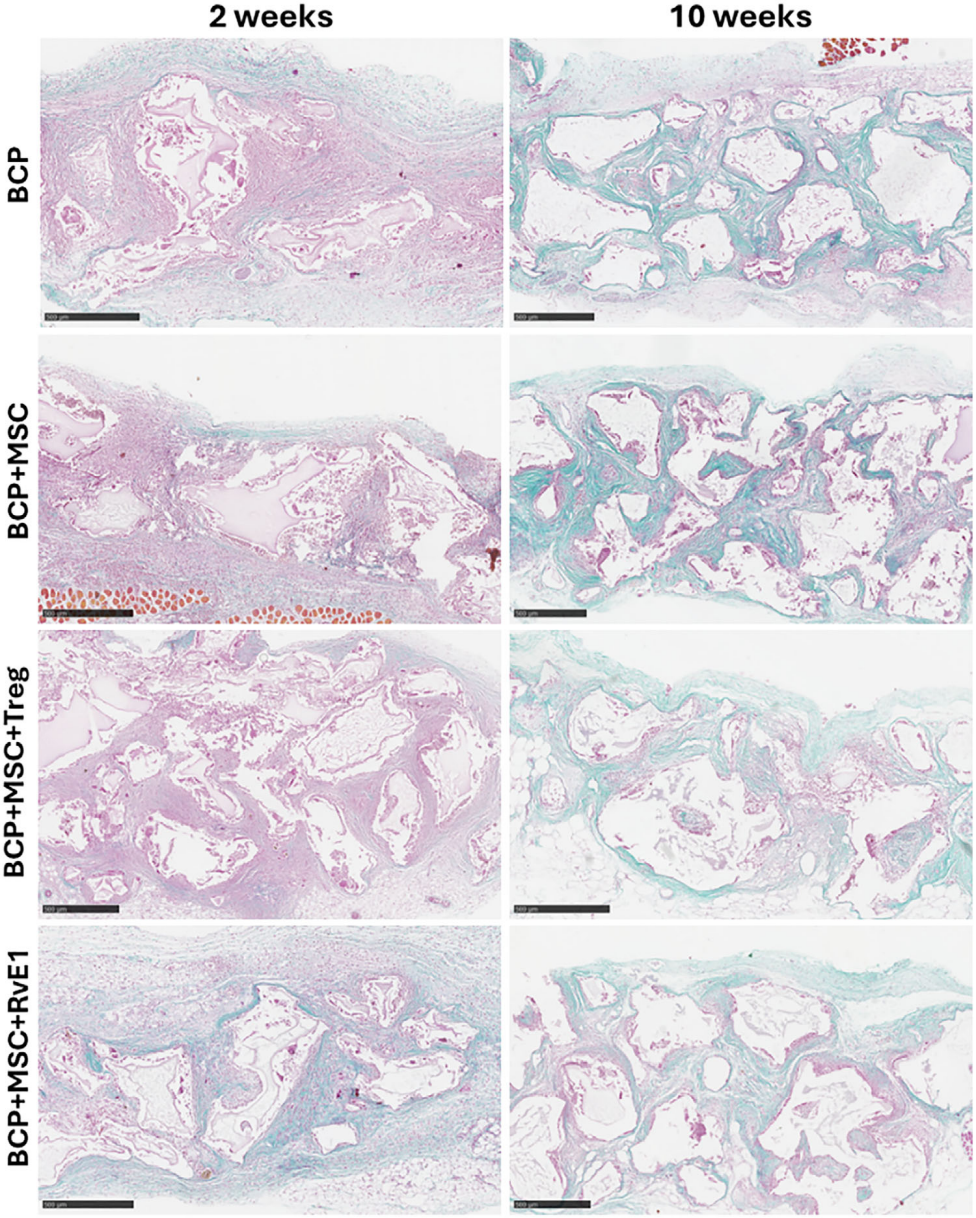
BCP+MSC+RvE1 exhibits higher collagen staining after 2 weeks. Representative sections stained with Masson's Trichome after 2 and 10 weeks. Collagen fibers appear blue. Scale bar = 1 mm.

Masson's trichome staining at 2 weeks revealed faint collagen deposition across all groups, primarily near the fibrous capsule and diffusely within the surrounding connective tissue. No notable differences were observed between the groups, except for BCP+MSC+RvE1, which revealed strong staining intensity at 2 weeks (Figure 6, blue stain). In contrast, at 10 weeks, substantial collagen stain was present in all groups (Figure 6, blue stain), with intense collagen deposition predominantly observed in the connective tissue surrounding the granules.

**FIGURE 7:**
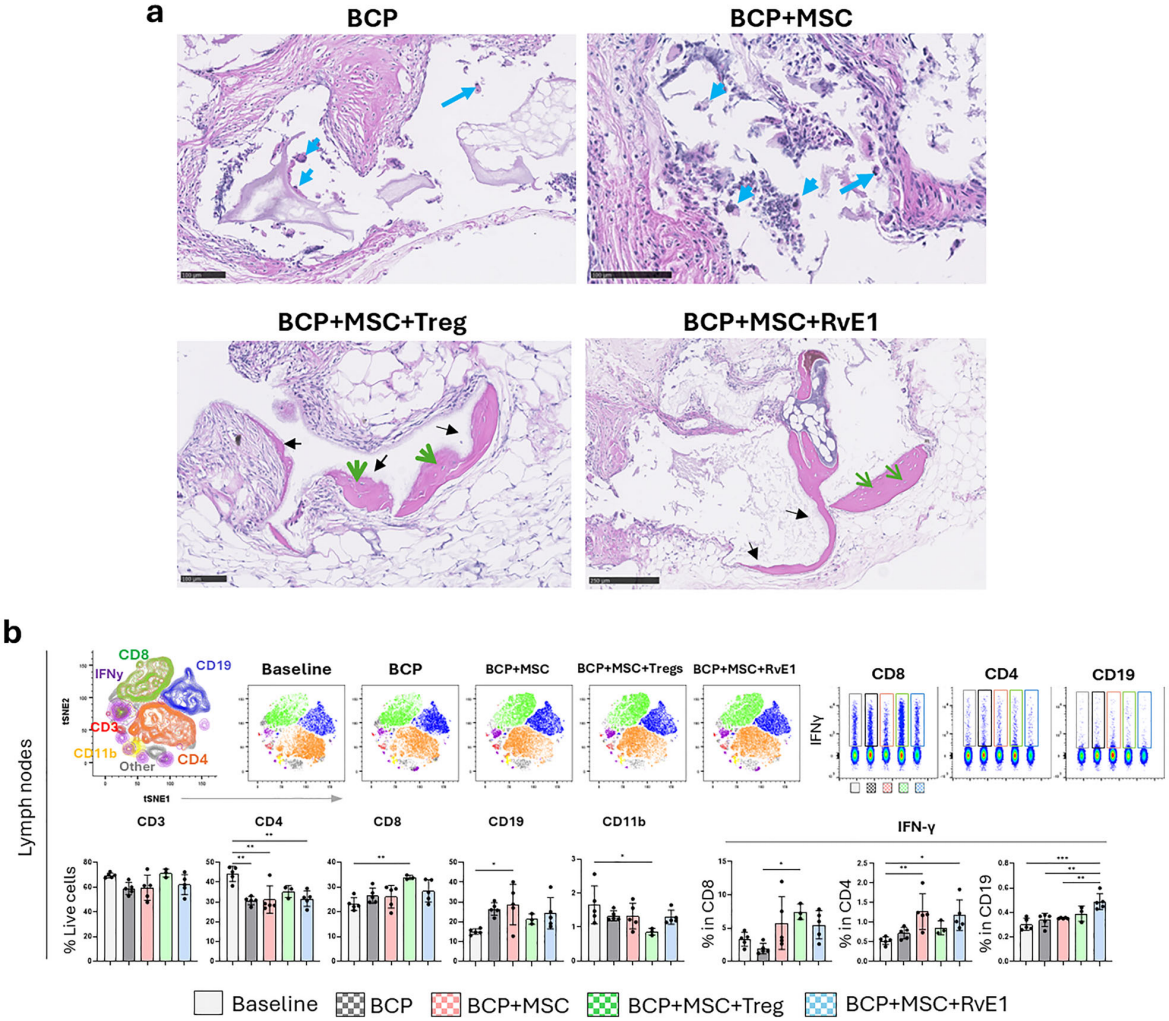
MSC priming strategies have divergent influences on immune cell recruitment and osteogenesis after 10 weeks. (a) Representative H&E‐stained histological sections after 10 weeks. Blue arrows indicate inflammatory cell infiltration, black arrows denote remaining BCP granules, and green arrows highlight mineralized bone forming at the edges of BCP granules. (b) Dimensional reduction using *t*‐SNE illustrating immune cell distribution, with distinct clustering patterns across groups. “Baseline” refers to untreated, construct‐free animals. Absolute quantification of each immune cell subset, along with absolute IFN‐γ production in lymph nodes, is shown. Data are presented as means +/– SEM (*n* = 3–5 animals per group). Statistical significance was determined by one‐way ANOVA with post hoc tests. *p < 0.05, **p < 0.01, ***p < 0.001.

The osteogenic potential of the implanted construct was evaluated by mineralization or ectopic bone formation after 2 and 10 weeks. Pronounced ectopic bone development was observed at 10 weeks in the BCP+MSC+Treg and BCP+MSC+RvE1 groups (Figure 7a), whereas no signs of mineralization were observed in either the BCP or BCP+MSC group. Mineralized tissue formation in these groups was observed to initiate along the periphery of BCP granules (Figure 7a, black arrows). This mineralized tissue exhibited woven bone characteristics, with interlacing and organized arrangements of the collagen fibers. Moreover, lacunae housing osteocytes were visible within the matrix (Figure 7a, green arrows). These mineralized regions corresponded to areas that, at 2 weeks, had been characterized by dense connective tissue surrounding the BCP granules. The BCP+MSC group continued to exhibit a higher abundance of inflammatory cells in the connective tissue surrounding the granules at 10 weeks compared to the other groups (Figure 7a, blue arrows). The nonmineralized groups, BCP and BCP+MSC, portrayed denser, more regular connective tissue, with reduced extracellular matrix and fibers arranged in parallel pattern at 10 weeks (Figure 7a).

### 2.4 Distinct Immune Cell Recruitment and Varied IFN‐γ Production After 10 Weeks in Regional Lymph Nodes and Spleen

For the absolute quantification of distinct immune cell populations in lymph nodes and spleen at the 10‐week time point, 3–5 animals were analyzed per group. In the lymph nodes, the BCP+MSC+Treg group demonstrated a higher count of CD8^+^ cytotoxic T cells compared to baseline animals (*p* = 0.005). Additionally, all groups showed a reduction in CD11b^+^ myeloid cells, with the lowest significant count observed in the BCP+MSC+Treg group (*p* = 0.037) compared to baseline animals (Figure 7b). BCP (*p* = 0.001) and BCP+MSC (*p* = 0.001) groups portrayed significantly lowest number of CD4^+^ T helper cells compared to baseline animals, while BCP+MSC exhibited the highest CD19^+^ B cell count compared to baseline animals (*p* = 0.025) (Figure 7b). At 10 weeks, lymph nodes showed a trend of increased IFN‐ɣ production by CD8^+^ cytotoxic T cells in the BCP+MSC+Treg (*p* = 0.025) compared to BCP. The highest IFN‐ɣ production from CD4^+^ T cells was observed in the BCP+MSC (*p* = 0.008) and BCP+MSC+RvE1 (*p* = 0.023) groups compared to baseline animals. Furthermore, CD19^+^ B cells showed elevated IFN‐γ production in the BCP+MSC+RvE1 group compared to baseline animals (*p* = 0.0001), BCP (*p* = 0.001), and BCP+MSC (*p* = 0.004) (Figure 7b).

In the spleen at 10 weeks, the BCP group showed significantly lower expression of CD4^+^ T helper cells compared to baseline animals (*p* = 0.041). The BCP+MSC+Treg group displayed the highest number of CD19^+^ B cells compared to the baseline animals (*p* = 0.002) and BCP+MSC (*p* = 0.017) (Figure S5). The BCP+MSC group demonstrated significantly higher IFN‐γ expression from CD4^+^ compared to baseline animals (*p* = 0.001) and BCP (*p* = 0.008). It also showed the highest IFN‐γ expression from CD19^+^ cells too compared to baseline animals (*p* = 0.001) and BCP (*p* = 0.016) (Figure S5).

### 2.5 Local and Systemic Immunomodulation In Vivo at the Protein Level Following MSC Priming via Coculture with Treg or RvE1

All analytes were detected in the implanted scaffold construct across all groups; however, not all were detected in the serum samples (Tables S2–S5).

**FIGURE 8:**
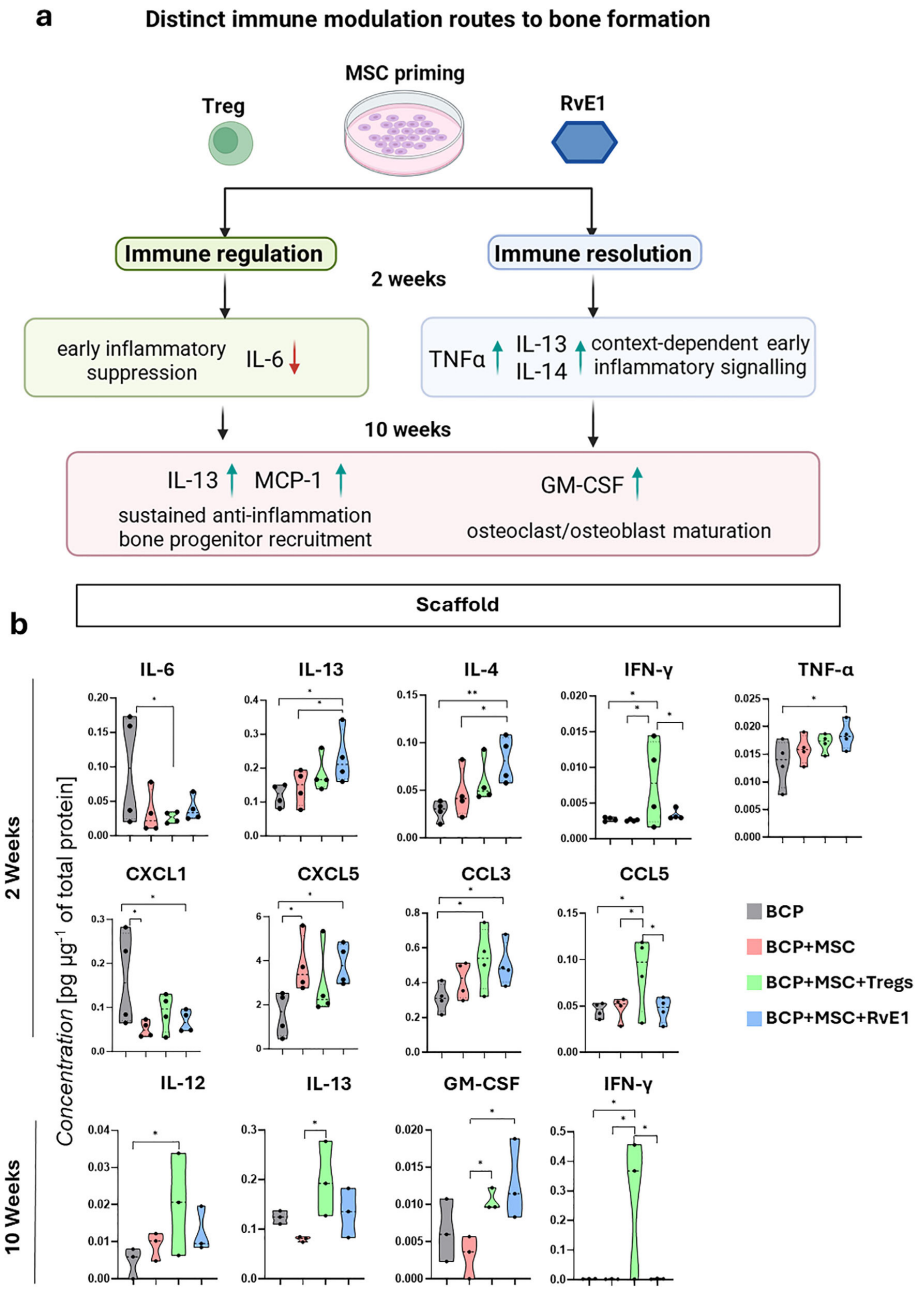
Immunomodulation locally at the protein level when MSC are primed with Treg coculture or RvE1. (a) Schematic summarizing distinct immunomodulation cytokines and chemokines induced by BCP+MSC+Treg and BCP+MSC+RvE1. Created with Biorender.com; used under license. (b) Quantification of significantly expressed cytokines and chemokines from scaffolds after 2 and 10 weeks. Data are presented as means +/– SEM, *n* = 4 animals per group at 2 weeks and *n* = 3 animals per group at 10 weeks. Statistical significance was determined by one‐way ANOVA with post hoc tests. *p < 0.05, **p < 0.01.

At 2 weeks, a total of 4 animals were analyzed. In the scaffold constructs, the proinflammatory interleukins, IL‐1α and IL‐2 were highly detected across all groups at 2 weeks, while IL‐6 was highly expressed exclusively in the BCP group and significantly downregulated in BCP+MSC+Treg compared to BCP (*p* = 0.042). Regarding prohealing/anti‐inflammatory markers in the constructs at 2 weeks, BCP+MSC+RvE1 and BCP+MSC+Treg exhibited the highest levels of IL‐13, with significance for BCP+MSC+RvE1 compared to BCP (*p* = 0.016) and BCP+MSC (*p* = 0.047). These groups also showed the highest levels of IL‐4, significant for BCP+MSC+RvE1 compared to BCP (*p* = 0.005) and BCP+MSC (*p* = 0.04) (Figure 8b).

Distinct expression levels of chemokines and cytokines were also observed at 2 weeks. The BCP+MSC+Treg group showed significantly higher expression of CCL3 compared to BCP (*p* = 0.028) and of CCL5 compared to BCP+MSC (*p* = 0.024). Both BCP+MSC+RvE1 and BCP+MSC exhibited similarly high expression of CXCL5 and low expression of CXCL1 compared to BCP (*p* = 0.019 and *p* = 0.042, respectively). TNF‐α was significantly more highly expressed in constructs from BCP+MSC+RvE1 compared to BCP (*p* = 0.032; Figure 8b). Notably, BCP+MSC+Treg exhibited significantly elevated IFN‐γ expression in the constructs at 2 weeks compared to BCP (*p* = 0.03), BCP+MSC (*p* = 0.026), and BCP+MSC+RvE1 (*p* = 0.050) (Figure 8b).

At 10 weeks, a total of 3 animals were analyzed. Analysis of pro‐ and anti‐inflammatory interleukins in the constructs showed that BCP+MSC+Treg had significantly increased IL‐12 compared to BCP (*p* = 0.045) (Figure 8b). Additionally, only the BCP+MSC+Treg group showed significantly higher IL‐13 expression compared to BCP+MSC (*p* = 0.013), representing the highest levels among all groups (Figure 8b).

After 10 weeks, constructs from BCP+MSC+Treg (*p* = 0.044) and BCP+MSC+RvE1 (*p* = 0.014) showed significantly increased granulocyte‐macrophage colony‐stimulating factor (GM‐CSF) expression compared to BCP+MSC, a marker that was absent in serum samples (Figure 8b). BCP+MSC+Treg also continued to exhibit significantly elevated IFN‐γ expression in the constructs at 10 weeks compared to all other groups (*p* = 0.024; Figure 8b).

Regarding serum levels, at 2 weeks, BCP+MSC+RvE1 showed significantly lower IL‐4 expression compared to BCP (*p* = 0.03; Figure S5). At 10 weeks, CXCL5 was significantly upregulated in BCP compared to BCP+MSC (*p* = 0.001) and BCP+MSC+RvE1 (*p* = 0.002). It was also significantly elevated in BCP+MSC+Treg compared to BCP+MSC (*p* = 0.007) and BCP+MSC+RvE1 (*p* = 0.013). The BCP+MSC+Treg group showed increased MCP‐1 expression compared to BCP (*p* = 0.034), BCP+MSC (*p* = 0.034), and BCP+MSC+RvE1 (*p* = 0.038) in serum. Both BCP+MSC+Treg (*p* = 0.001) and BCP+MSC+RvE1 (*p* = 0.013) showed significantly decreased CXCL9 compared to BCP+MSC (Figure S5). Finally, IFN‐γ expression was higher in the serum of the BCP+MSC+Treg group compared to BCP at 10 weeks (*p* = 0.043; Figure 8b).

### 2.6 Treg Depletion Modulates the Immune Response and RvE1 Therapy Improves the Regeneration Process

**FIGURE 9:**
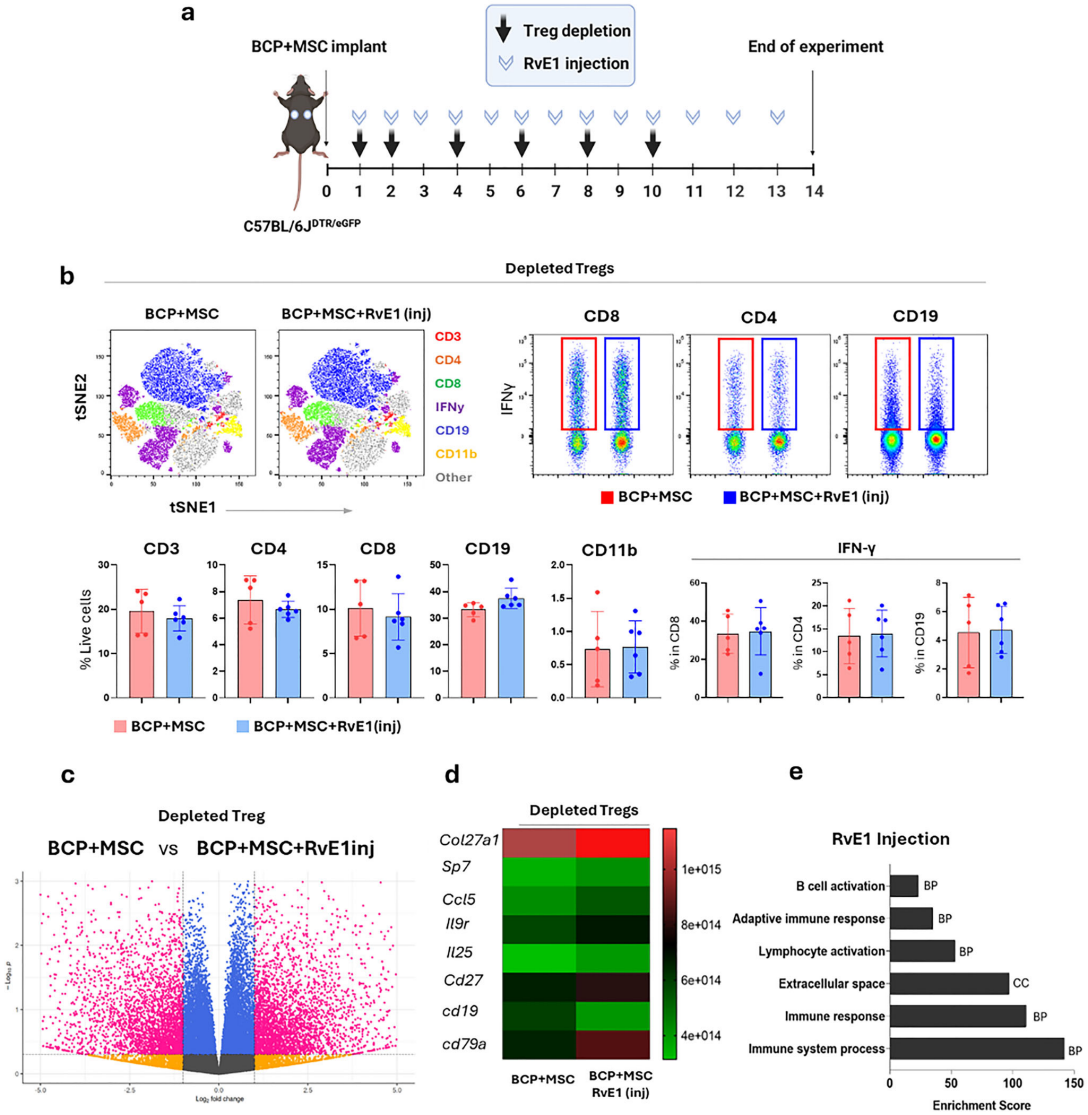
Treg depletion modulates the immune response and RvE1 therapy impacts the regeneration process. (a) Schematic summary of experimental design and groups used in the Treg‐depletion experiment. Created with Biorender.com; used under license. (b) Dimensional reduction using *t*‐SNE of immune cells from lymph nodes after 2 weeks, illustrating distinct population distributions across groups. Absolute quantification of each immune cell subset and corresponding IFN‐γ production is shown. Data are presented as mean +/– SEM (*n* = 5–6 animals per group). Statistical significance was determined by independent sample *t*‐test. (c) Volcano plot showing transcriptional expression differences between (dep)BCP+MSC and (dep)BCP+MSC+RvE1inj. *N* = 4 animals per group (d) Selected significantly expressed genes related to osteogenesis and immune regulation. *N* = 4 animals per group. (e) Enrichment scores identifying functional modules in (dep)BCP+MSC+RvE1inj. *N* = 4 animals per group.

Our objective was to investigate the impact of reduced Treg levels in vivo on the ectopic regeneration induced by BCP+MSC and to gain an insight into how specialized proresolving lipid mediators may compensate for Treg absence in this context (Figure 9a).

A total of 5–6 animals were analyzed per group for absolute quantification of immune cell populations in lymph nodes. Although there were noticeable trends after 2 weeks of local administration of RvE1 in the implanted BCP+MSC construct ((dep)BCP+MSC+RvE1inj) group, such as decreased CD8^+^ cytotoxic cells and increased CD19^+^ B cells, these changes were not statistically significant (Figure 9b). No significant differences were observed IFN‐ɣ production across cell types (Figure 9b).

We further investigated the transcriptional changes during Treg depletion by comparing (dep)BCP+MSC and (dep)BCP+MSC+RvE1inj, analyzing 4 animals per group. A total of 607 genes were upregulated and 627 downregulated in (dep)BCP+MSC+RvE1inj (Figure 9c). Local administration of RvE1 resulted in significant upregulation of bone formation‐related genes, *Col27α1* (*p* = 0.0032) and *Sp7* (*p* = 0.013). Genes related to immunomodulation and anti‐inflammation were also significantly expressed in this group, including *Ccl5* (*p* = 0.0087), *Il9r* (*p* = 0.017), and *Il25* (*p* = 0.028; Figure 9d). Interestingly, (dep)BCP+MSC+RvE1inj also expressed markers related to B cell recruitment and activation, including *CD27* (*p* = 0.0067), *CD19* (*p* = 0.03), and *CD79a* (*p* = 0.0036; Figure 9d). Enrichment analysis revealed pathways characteristic of adaptive immune responses, particularly B cell activation among other pathways in (dep)BCP+MSC+RvE1inj (Figure 9e).

**FIGURE 10:**
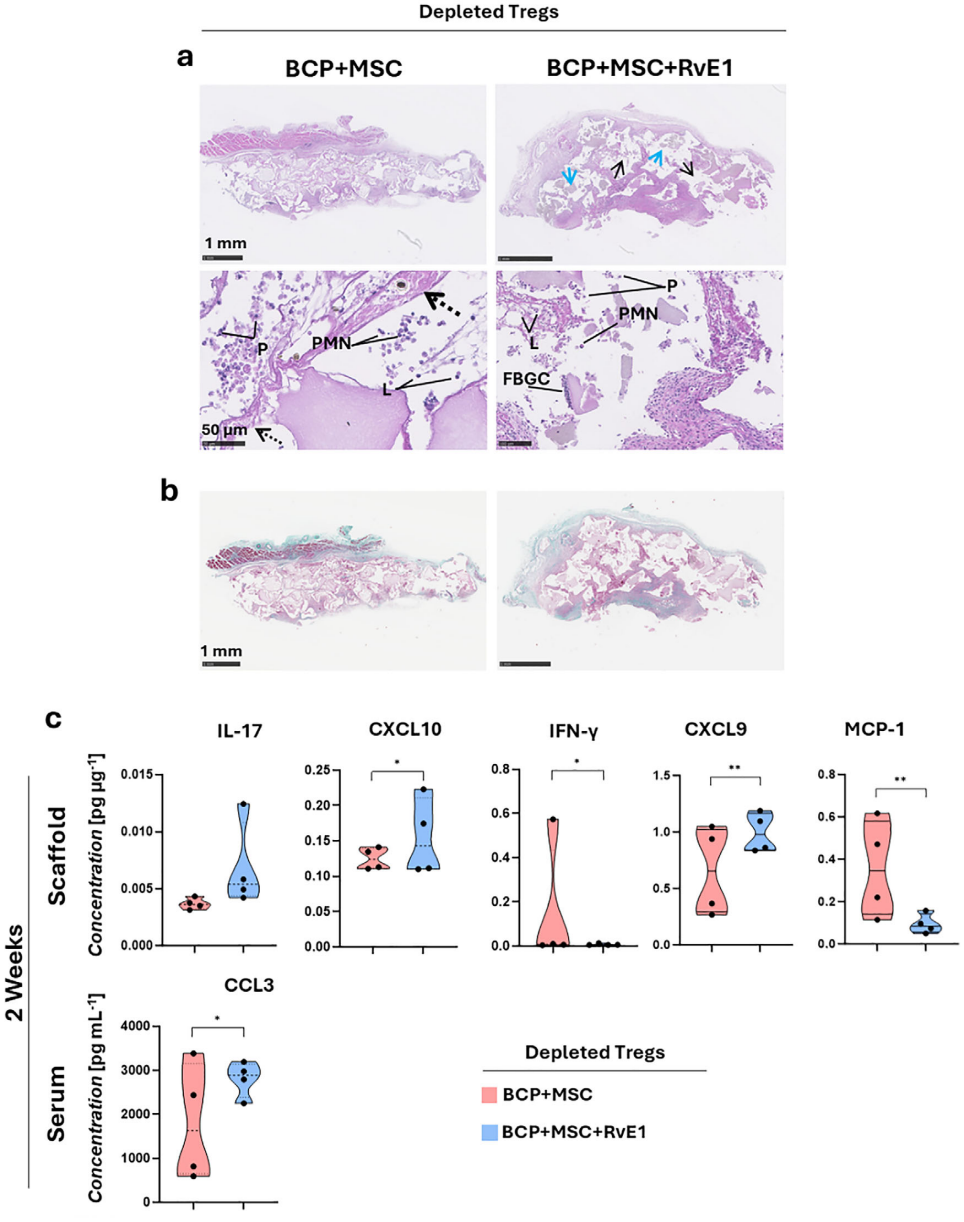
Histological and protein variations after Treg depletion and RvE1 therapy. (a) Representative H&E‐stained sections after 2 weeks. Low magnification images (scale bar = 1 mm) show BCP granules (blue arrows) and discontinued infiltrating connective tissue in (dep)BCP+MSC+RvE1inj (black arrows). Higher magnification images (scale bar = 50 µm) highlight loose connective tissue with irregular string‐like fibers in (dep)BCP+MSC (dotted black arrows). P: Plasma cells, L: Lymphocytes, PMN: polymorphonuclear cells, FBGC: foreign body giant cells. (b) Representative Masson's Trichome‐stained sections after 2 weeks. Collagen fibers appear blue. Scale bar = 1 mm. (c) Quantification of significantly expressed cytokines and chemokines from scaffolds and serum after 2 weeks. Data are presented as mean +/– SEM (*n* = 4 animals per group). Statistical significance was determined by independent sample *t*‐test, *p < 0.05, **p < 0.01.

Histologically, in the absence of Treg, the (dep)BCP+MSC+RvE1inj group exhibited denser connective tissue infiltration around the granules at 2 weeks. However, this connective tissue displayed discontinuities (Figure 10a, black arrows), in contrast to observations in nondepleted animals. The granules also appeared to undergo breakdown (Figure 10a, blue arrows), and the intermittent connective tissue may reflect disruption from local injections of RvE1. Both groups displayed inflammatory infiltration, although it was more dispersed in the (dep)BCP+MSC group, where cells also appeared smaller. This group exhibited loose connective tissue characterized by irregular string‐like fibers (Figure 10a, dotted arrows), and limited extracellular matrix or fibroblast activity. Both groups contained polymorphnuclear cells, lymphocytes, and plasma cells, but in (dep)BCP+MSC group these inflammatory cells were scattered and not consistently localized near granules. Giant cells were identified only near granules in (dep)BCP+MSC+RvE1inj (Figure 10a,b), although fewer than in constructs from healthy mice. The (dep)BCP+MSC+RvE1inj group also displayed more collagen staining in Masson's trichome at 2 weeks (Figure 10b).

Treg depletion and local RvE1 administration affected cytokine expression in both constructs and serum (Figure 10c). After 2 weeks (*n* = 4 animals), (dep)BCP+MSC+RvE1inj exhibited increased IL17 levels in both the scaffold construct and serum. Similar to healthy animals, CXCL5 was the most prominently expressed chemokine in constructs of Treg‐depleted mice, while CCL3 was the highest in serum (Figure 10c). The localized administration of RvE1 resulted in significantly higher secretion of CXCL10 (*p* = 0.021) and CXCL9 (*p* = 0.002) in constructs, and CCL3 (*p* = 0.012) in the serum. Under Treg‐depleted conditions, IFN‐γ was significantly higher in (dep)BCP+MSC constructs compared to (dep)BCP+MSC+RvE1inj constructs (*p* = 0.025). Furthermore, RvE1 delivery also significantly reduced MCP‐1 (*p* = 0.007) production in constructs (Figure 10c).

To investigate the effect of Treg depletion on the osteogenesis in BCP+MSC constructs, we compared the transcriptome of (dep)BCP+MSC with that of BCP+MSC from 4 animals. When normalized to BCP+MSC, (dep)BCP+MSC showed upregulation of 1727 genes and downregulation of 1893 genes. Interestingly, (dep)BCP+MSC expressed an upregulation in an osteoblast marker, *Bglap3* (*p* = 0.039). On the other hand, BCP+MSC upregulated the collagenous and noncollagenous bone matrix genes, including *Col1α1* (*p* = 0.0005), *Col1α2* (*p* = 0.0001), *Sp7* (*p* = 0.01), and *Ibsp* (*p* = 0.00 002). In (dep)BCP+MSC, a proinflammatory profile with lymphocyte activation was observed, with significant upregulation of *Il17F* (*p* = 0.003), *Ctla4* (*p* = 0.004), *Slamf1* (*p* = 0.005), *Icos* (*p* = 0.005), and *CD8b1* (*p* = 0.018). Enrichment analysis showed pathways related to chondrogenesis, monocyte development, and extracellular organization among other pathways enriched in BCP+MSC when normalized to (dep)BCP+MSC (Figure S4a,b).

## 3 Discussion

Striking the right balance between initiating proinflammatory responses and enhancing anti‐inflammatory signaling is crucial, as both contribute to orchestrating efficient bone regeneration. The most common strategy for modulating immune cell recruitment after injury has involved the local delivery of immunomodulating molecules via implanted biomaterials [25, 26]. Recently, evidence demonstrating the adoptive transfer of immune‐regulatory cells, such as Treg has shown a significant acceleration of bone healing, as well as healing in other tissues, when compared to transfer of conventional CD4^+^ T cells [27].

We demonstrated that coculturing MSC with Treg or priming with RvE1 prior to implantation induces local and systemic immunomodulation in vivo. However, the two priming strategies modulate the immune system in distinctive ways, while both promote a prohealing environment and facilitate bone formation. Absolute total cellularity increased in both BCP+MSC+Treg and BCP+MSC+RvE1 groups from 2 weeks onward in lymphoid organs, indicating active immune modulation driven by these two priming strategies. An early increase in immune cell numbers within the defect region has previously been shown to correlate with the therapeutic efficacy of the implanted biomaterial [28].

A closer look at the cell‐type‐specific cellularity revealed a significant reduction in CD8^+^ cytotoxic T cells in both lymphoid organs of the BCP+MSC+Treg group from 2 weeks onward. Previous reports have shown that depletion of CD8^+^ T cells in a mouse osteotomy model enhances endogenous fracture regeneration, whereas adoptive transfer of CD8^+^ T cells impairs healing [29]. Evidence from both preclinical and clinical studies indicates that bone regeneration is compromised by the local accumulation of CD8^+^ effector T cells. This impairment is attributed to a shift from a beneficial early inflammatory response to a more detrimental inflammatory state. Adoptive transfer of Treg was found to counteract this undesired inflammation [5]. Therefore, the early reduction in cytotoxic T cells observed in the BCP+MSC+Treg group may contribute to mitigating excessive inflammation that support enhanced healing and bone formation. Furthermore, the significant upregulation of CD8^+^ T cells in this group at 10 weeks may reflect a role in bone remodeling once osteogenesis is established.

No reduction of CD8^+^ T cells was observed in the BCP+MSC+RvE1 group at 2 weeks, despite evidence of bone formation by 10 weeks, suggesting a distinct mechanism of action. SPMs exert diverse effects on myeloid cells, including regulation of cytokine production and inhibition of activation and maturation [25]. GM‐CSF levels were significantly increased in BCP+MSC+RvE1 constructs at 10 weeks. GM‐CSF is recognized to have dual roles, both pro‐and anti‐inflammatory [30], while functioning as a growth and differentiation factor for macrophages and enhancing osteoclastogenesis [31]. Osteoclasts play a crucial role in the bone healing cascade, and the presence of osteoclasts at calcium phosphate implant sites has been shown to precede new bone formation [32]. Therefore, the regulatory effects of GM‐CSF in this group may depend on the presence of other cytokines, such as TNF‐α and IL‐17, with TNF‐α significantly elevated in BCP+MSC+RvE1 constructs at 2 weeks. The synergistic effects of IL‐17A and TNF‐α enhance osteogenic differentiation and mineralization of human MSC by down‐regulating Dickkopf‐1, a Wnt‐signaling pathway inhibitor [33]. IL‐17A has also been shown to increase osteoblastic differentiation of murine calvaria osteoblasts through upregulation of osteogenic genes [34, 35]. Furthermore, IL‐17A promotes GM‐CSF production from osteoblasts, which subsequently reduces Receptor Activator of Nuclear Factor kappa‐B (RANK) expression in osteoclast precursors and inhibits osteoclastogenesis. This may explain why elevated GM‐CSF levels were observed only at 10 weeks, corresponding to remodeling phases [36].

The group BCP+MSC+Treg group displayed significantly high MCP‐1 levels in serum at 10 weeks, whereas other groups showed little or no production. MCP‐1, also known as CCL2, is a strong chemoattractant for MSC and orchestrates the early inflammatory phase of bone healing by recruiting monocyte‐derived cells that release pro‐osteogenic cytokines [37, 38]. This highlights one mechanism through which Treg fine‐tune macrophage responses, promoting a shift toward a healing supportive phenotype [27].

We focused on IFN‐γ release by distinct immune subsets due to its pleiotropic roles in innate and adaptive immunity. IFN‐γ has also been implicated in bone metabolism, although [39, 40] with conflicting pro‐ and anti‐osteogenic context dependent reports. Emerging evidence points to potential dose‐ or time‐dependent effects of IFN‐γ on bone progenitor cells, where it can exert either stimulatory or inhibitory actions depending on the cellular and microenvironmental conditions [41]. In our results we observed a reduced IFN‐γ–producing CD4^+^ cells in local lymph nodes and reduced IFN‐γ–producing CD8^+^ T cells systemically (spleen) at 2 weeks in the BCP+MSC+Treg group, with suppression of CD4^+^ T IFN‐γ continuing at 10 weeks. On the contrary, CD8^+^ T cells showed the highest IFN‐γ production at 10 weeks. Previous reports [41] suggest that IFN‐γ may inhibit osteoblast differentiation early during bone formation but promote it later. This may explain why we observed lower IFN‐γ production at 2 weeks.

Exogenous Treg reduce neutrophil and cytotoxic T‐cell accumulation and IFN‐γ production in damaged tissues, dampening proinflammatory macrophage phenotypes [27]. A major influence of local Treg in early muscle injury is to suppress IFN‐γ production by CD4^+^ T helper and CD8^+^ T cells [42], which may have occurred in our animals. Multiplex and transcriptomic data showed significantly increased total IFN‐γ in BCP+MSC+Treg constructs at both time points. IFN‐γ inhibits fibroblast proliferation under fibrotic conditions, indirectly favoring bone formation. Indeed, we observed reduced fibrous capsule thickness in the BCP+MSC+Treg group at both time points. Although this was total IFN‐γ production, it still plays a function in endogenous Treg expansion, supporting an immunosuppressive and healing environment. Previous reports demonstrated higher levels of IFN‐γ in subsets of acute myeloid leukemia patients, where IFN‐γ upregulated immunosuppressive genes in MSC and expands Treg through indoleamine 2,3‐dioxygenease 1 (IDO1) [43]. MSC can also induce a Treg subpopulation, that secretes IFN‐γ and IL‐10 [44]. These type 1 Treg are induced by IFN‐γ and IL‐12 [45], and function early during inflammation representing the first line of Treg that suppresses initial immune responses. In line with this, IL‐12 levels were significantly elevated at 10 weeks in the BCP+MSC+Treg group.

IL‐6 and IFN‐ɣ are multifunctional cytokines that often act in opposition when regulating immune responses and cell proliferation [46]. BCP+MSC+Treg showed significantly low IL‐6 levels at 2 weeks, corroborated with significantly lower *Il6* gene expressions in this group. These high IFN‐γ and low IL‐6 levels suggest a Th1‐skewed rather than a Th17 response, coupled by low IL‐17 protein levels and reduced CD8^+^ cells, which may be favorable for controlled inflammatory profile and potentially immune‐regulated regeneration. Indeed, anti‐inflammatory IL‐4 and IL‐13 were also elevated early in BCP+MSC+Treg constructs, with IL‐13 remaining significantly high up to 10 weeks. This pattern underscores sustaining these canonical type 2 cytokines to augment osteogenic differentiation and contribute to bone matrix formation. IL‐4 and IL‐13 promote M2 (anti‐inflammatory/prohealing) macrophage polarization, which is associated with an anti‐inflammatory and proregenerative microenvironment [47]. IL‐4 suppresses RANKL‐induced osteoclast differentiation through direct action on osteoclast precursors [48]. Recently, Palmqvist et al. [49] reported both IL‐4 and IL‐13 inhibit osteoclast differentiation by a mechanism that increases osteoprotegerin (OPG) in osteoblasts and also decreases RANKL and RANK expression. Moreover, macrophages polarized by IL‐4 and IL‐13 modulate fibroblast proliferation and endothelial cell activity, which are essential for neovascularization and de novo bone formation [50]. Our results align with IL‐4/IL‐13‐driven immune environments conducive to bone regeneration.

In BCP+MSC controls, T cell IFN‐γ production remained high from 2 to 10 weeks, corresponding with no bone formation. The proinflammatory IFN‐γ and TNF‐α, at the beginning of an injury determines the healing process. However, the timely termination of this proinflammatory process is a prerequisite for the initiation of later regenerative phases in osteogenesis. High levels of IFN‐γ can inhibit MSC osteogenesis, likely through synergy with TNF‐α, by inducing MSC apoptosis and inhibiting the Runx‐2 pathway [12]. Notably, reducing IFN‐γ and TNF‐α via Treg infusion improved MSC‐mediated bone repair in mice [12]. In the BCP+MSC+RvE1 constructs, TNF‐α was significantly elevated at 2 weeks, but the absence of IFN‐γ may explain the successful bone formation. RvE1 can regulate IFN‐ɣ production, contributing to the overall reduction of inflammation [51]. Indeed, local RvE1 reduced IFN‐ɣ production in (dep)BCP+MSC+RvE1inj constructs compared to Treg‐depleted controls. Interestingly, this was consistent with the reduction in total IFN‐ɣ production in constructs of MSC primed with RvE1 in healthy animals. These findings along with previous reports support the context‐, dose‐, and stage‐dependent effects of IFN‐γ in bone.

B cells are recognized modulators of bone remodeling, through cytokine regulation and OPG production, which inhibits osteoclastogenesis [52]. We observed increased CD19^+^ B‐cell frequency in lymph nodes of BCP+MSC+Treg group as early as 2 weeks, suggesting a potential alternative immunoregulatory mechanism. B cell activation and maturation are supported by IL‐4 [53], which was robustly expressed by BCP+MSC+Treg constructs at both transcriptomic and proteomic levels. Supporting the functional role of CD19^+^ B cells in bone homeostasis, previous studies using CD19‐knockout mice have demonstrated enhanced osteoclast activity and bone resorption, thereby reinforcing the protective role of these cells in skeletal integrity. Consistently, following Treg depletion, we observed a similar (albeit insignificant) pattern of CD19^+^ B‐cell accumulation in (dep)BCP+MSC+RvE1inj lymph nodes, supported by significant upregulation of *CD19* gene and genes associated with B‐cell chemotaxis and activation. Thus, this protective mechanism can also be postulated in this study. Recent work by Zhang et al. [54] employing single‐cell analysis highlight the temporal dynamic B‐cell involvement in fracture repair. Their data suggest that during early callus formation, a reduction in B‐cell numbers correlates with increased osteoblast and osteoclast activity, whereas the subsequent healing phase is characterized by B cell‐mediated modulation of osteoblast differentiation. Although our observations in lymph nodes and spleen do not entirely replicate this temporal pattern, likely due to differences in anatomical locations or experimental context, they underscore the emerging role of B‐cells as immunological regulators of tissue regeneration and bone remodeling, and suggest a potential immunoregulatory interplay between Treg and B‐cells in tissue regeneration. Additionally, Moore et al. [55] demonstrated that B cell responses to biomaterial implantation in muscle injuries elicit a range of local, regional, and systemic immune alterations. This further supports the premise that B cells may play a broad role in orchestrating the immune response to regenerative stimuli, including those induced by biomaterials.

No differences were found in bone quality between BCP+MSC+Treg and BCP+MSC+RvE1, while immune cells persisted in BCP and BCP+MSC constructs at 10 weeks. This highlights the osteo‐immunomodulating effects of priming MSC with either Treg or RvE1. BCP+MSC alone produced no bone, in line with Liu et al. [12], who reported that MSC with calcium phosphate particles induced ectopic bone in immunodeficient mice but not in immunocompetent C57BL/6 mice.

Distinct osteogenic transcriptional profiles were revealed when comparing MSC primed with Treg or RvE1. In BCP+MSC+Treg, genes associated with endochondral ossification were modulated, including upregulation of *Scrg1*, and downregulation of *Matn3* and *Has2*. *Scrg1*, a secreted protein involved in MSC recruitment and tissue regeneration [56], is implicated in enhancing chondrogenic differentiation, and in preserving the osteogenic potential of MSC even after extended expansion [57]. The downregulation of *Matn3* and *Has2*, suggests loss of cartilage extracellular matrix and a shift toward ossification. Lineage‐tracking studies show hypertrophic chondrocytes can transdifferentiate into osteoblasts, directly contributing to bone formation during development, fracture healing, and ectopic ossification [58]. Supporting this, we observed a marked increase in *Ibsp*, a key bone matrix marker, in the BCP+MSC+Treg group, indicating a transition toward endochondral ossification within the ectopic bone model.

In contrast, BCP+MSC+RvE1 exhibited upregulation of genes more consistent with bone development, such as *Nog* [59], and osteoblast maturation, including, *Bglap*, and *Fgf4*. *Bglap*, a calcium‐binding protein secreted by mature osteoblasts, is enhanced by *Fgf4* signaling [60], underscoring a distinct osteoblast maturation pathway in the BCP+MSC+RvE1 group.

Comparing BCP+MSC+Treg and BCP+MSC+RvE1 transcriptome revealed remarkable upregulation of amphiregulin (*Areg)*, a gene exclusively found in the BCP+MSC+Treg group. *Areg* is a key immunoregulatory molecule at the interface of immune response and tissue regeneration. Following injury, it is secreted by multiple immune cell subsets, including Treg, and was shown to attract Treg to the injury site. In skeletal muscle, *Areg*‐mediated signaling supports myogenic progenitor cell expansion, a function that becomes compromised in the absence of Treg but can be restored with exogenous *Areg* [8]. *Areg* during the repair phase, acts on tissue‐resident progenitor cells through Epidermal Growth Factor Receptor (EGFR)‐dependent pathways to enhance their survival, proliferation, and differentiation [61, 62]. *Areg's* actions are not limited to immune modulation but extend to direct cellular cross‐talk with tissue‐specific stem cells, making it a central effector of the proregenerative capacity of Treg, and thus highlighting the enhanced regenerative capacity of MSC cocultured with Treg [30].

Importantly, Wu et al. [63] demonstrated in a murine fracture model that bone‐callus Treg overexpress Areg protein and that Areg promotes proliferation and differentiation of osteogenic precursor cells. Moreover, they also showed that Areg can reverse compromised fracture healing due to Treg deficiency [63]. While the role of *Areg* in maintaining bone homeostasis is well established [64], only emerging evidence links Treg‐derived Areg to enhanced osteogenesis during repair. Additionally, macrophage‐derived *Areg* can enhance the osteogenic differentiation of chondrocytes [65], highlighting its broader relevance in skeletal repair. Based on these findings, we propose that the upregulation of *Areg* observed specifically in the BCP+MSC+Treg group, and not in BCP+MSC+RvE1, may reflect Treg‐mediated signaling, suggesting a potential, yet unexplored, role for Treg–*Areg* interactions in promoting MSC‐based bone regeneration.

To investigate endogenous Treg in MSC‐based regeneration in vivo, we used a Treg‐ablation model, coupled with a proresolving lipid mediator to restore homeostasis. RvE1 has a very short half‐life in vivo due to rapid metabolic inactivation, limiting its local persistence unless administered repeatedly [66]. Previous studies consistently use daily or frequent dosing to maintain its proresolving activity [24, 67]. Treg depletion creates a highly inflammatory environment, further necessitating sustained exposure. Therefore, daily local application was chosen to compensate for rapid clearance and maintain resolution signals. This schedule is supported by our prior work showing that 1 µM RvE1 has a preventive role in the establishment of the adaptive immune response during inflammation, and bone protective capacity in periodontal bone loss models [24].

RvE1 limits inflammation through several mechanisms, including immune cell recruitment [24]. In line, we have seen, albeit insignificantly, that local delivery of specialized proresolving lipid mediator alters immune cell recruitment. Chemokines predominately promoting immune cell recruitment and contributing to early tissue remodeling, such as CXCL9 and CXCL10 were found to be significantly upregulated in (dep)BCP+MSC+RvE1inj constructs, confirming their immunoregulatory impact. Histology was assessed at 2 weeks because Treg‐depleted animals could not be kept longer, as they developed marked, escalating inflammation. However, enhanced extracellular matrix organization could be observed in (dep)BCP+MSC+RvE1inj, supported by increased expression of the *Col27a1* gene, a fibrillar collagen involved in matrix assembly and skeletal development. This underscores the role of Treg in collagen deposition, an effect previously reported in tumor microenvironment models [68]. The lack of FBGC in the depleted group was an interesting phenomenon, likely due to combined effects of altered immune cell polarization, impaired macrophage fusion, a dysregulated inflammatory environment, and impaired tissue healing. Macrophages, particularly M2‐like, are critical for FBGC formation. Treg have been shown to promote the differentiation of macrophages toward M2 phenotypes as part of their tissue‐healing mechanisms in various tissues [27].

We and others have previously demonstrated that RvE1 attenuates IL‐17 production during bone homeostasis, specifically by regulating Th17 cell function [24, 69]. In this study, we observed that delivering RvE1 in a Treg‐depleted environment resulted in production of IL‐17 in the implants, albeit insignificant. Nevertheless, we can postulate that RvE1 immunomodulation may have promoted IL‐17 production from other immune cell compartments as a mechanism to accelerate prohealing responses. Indeed, previous reports have shown that early IL‐17 expression is prohealing, and an in vivo study demonstrated the crucial role for IL‐17‐producing gamma delta T cells in accelerating bone formation by stimulating MSC proliferation and osteoblastic differentiation [70]. These assumptions are corroborated by the significantly high expression of *Sp7* in (dep)BCP+MSC+RvE1inj, a key osteogenic transcription factor.

Despite the distinct immunomodulatory mechanisms by which Treg and RvE1 priming enhance MSC‐mediated bone regeneration, our findings underscore that, despite divergent upstream pathways, both priming strategies converge on conserved modulators that integrate immune regulation with sequential bone‐healing cascades. Key signatures identified, Areg, GM‐CSF and IL‐13, are not only markers of distinct immune modalities but also critical effectors within canonical phases of bone repair. Areg signals through EGFR to accelerate callus formation and osteoblast development, enhancing progenitor survival and osteogenic differentiation, aligning with endochondral ossification [63]. Similarly sustained IL‐13 elevation up to 10 weeks reinforces the bone formation phase by suppressing osteoclastogenesis, and promoting extracellular matrix deposition, essential for transitioning to osteoid formation [49]. The late rise of GM‐CSF in RvE1 constructs corresponds to the remodeling phase, where GM‐CSF orchestrates osteoclast–osteoblast coupling, and its interplay with early TNF‐α likely facilitates osteoblast differentiation while tempering remodeling [36, 50].

Despite its strengths, the study has certain limitations. The short observation window in the Treg‐depletion model restricted long‐term assessment of bone formation. Importantly, the ectopic implantation model may not fully recapitulate the complex cellular and mechanical environment of orthotopic bone healing, including bone‐marrow derived progenitors, vascularization patterns, and load‐bearing dynamics. Additionally, although histology confirmed the presence of mature bone, we did not perform functional or quantitative bone metrics. Another limitation is the absence of human MSC validation, which constrains direct translational relevance. Future studies should incorporate human cells and orthotopic models to better predict clinical outcomes. Finally, although our immune profiling provided valuable insights, it did not encompass all immune subsets. This reflects a focused scope, and future studies using single‐cell approaches could further refine these mechanistic insights. Another limitation is that tri‐lineage differentiation of MSC was not performed; however, this was based on previously validated protocols and prior evidence confirming the multipotency of C57BL/6‐derived MSC using the same isolation and characterization methods, which consistently express Sca‐1. Despite these limitations, the study offers compelling mechanistic insight into distinct immunomodulatory pathways and highlights promising strategies for immune‐driven bone regeneration.

## 4 Conclusions

Taken together, this study demonstrated that priming MSC with either Treg or RvE1 enhances bone regeneration through distinct immunomodulatory mechanisms. Treg priming promoted a regulated anti‐inflammatory environment characterized by early reduction in IL‐6, a sustained IL‐13 elevation up to 10 weeks, early suppression of cytotoxic T cells and IFN‐γ production by CD4^+^ T cells in lymph nodes, reduced fibrous capsule, and an early increase in B‐cells, supporting ossification as reflected by upregulation of *Scrg1* and *Ibsp*. RvE1 priming, in contrast, leveraged early TNF‐α signaling and a late rise in GM‐CSF to promote osteoblast maturation, reflected by increased *Bglap*, despite absence of IFN‐γ production. Transcriptomic profiles confirmed divergent osteogenic pathways, with amphiregulin uniquely upregulated in the BCP+MSC+Treg group compared to BCP+MSC+RvE1, suggesting a novel Treg‐Areg axis in MSC‐based bone regeneration. Importantly, RvE1 retained partial immunomodulatory capacity even in Treg‐depleted environments. We therefore highlight the versatility of the immune system in directing osteogenesis and underscore the potential of tailoring immune‐driven strategies to optimize regenerative outcomes.

## 5 Experimental Section

### 5.1 Isolation and Expansion of Mouse Bone Marrow‐Derived Mesenchymal Stromal Cell and Regulatory T Cells

Primary mouse bone marrow‐derived MSC were isolated from the bone marrow of 8‐week‐old wild‐type C57BL/6J mice (The Jackson Laboratory, USA). The femur and tibia were dissected and bone marrow was flushed out using culture medium composed of alpha minimum essential medium (α MEM, Thermo Fischer Scientific, USA) supplemented with 10% FBS and 1% penicillin/streptomycin (Sigma‐Aldrich, USA). Cells were centrifuged and the cell pellet was directly seeded, without filtering or counting, into 60 mm culture plates, with all cells collected from each animal plated together. Cultures were maintained at 37°C in a 5% CO_2_ incubator, preserving the native niche with minimal disturbance for optimum growth [71]. Nonadherent cells were removed after 72 h, and cells were subcultured (1:2) at 90% confluence. MSC were phenotyped at passage 2. Cells were resuspended in PBS + 5% FBS and stained with the following antibodies: anti‐CD105, anti‐CD73, anti‐CD11b, anti‐CD45, anti‐CD44, anti‐CD29, and anti‐SCA1 (all from Biolegend, USA; Table S1). Flow cytometry was performed using a FACScan (Becton Dickinson, USA) and acquiring at least 2 × 10^5^ events per sample. Unstained and isotype controls were included. Data were analyzed using FlowJo software (v10.7.1, USA) (Figure S1a). The marker panel reflected the murine equivalent of the International Society for Cell and Gene Therapy (ISCT) minimal criteria for MSC characterization, adapted to include species‐specific markers [71].

Treg were isolated from Foxp3^DTR/eGFP^ (The Jackson Laboratory, USA; Strain#: 016958). These mice express diphtheria toxin receptor (DTR) and enhanced green fluorescent protein (eGFP) under the *Foxp3* transcription factor gene. Single‐cell suspensions were prepared from spleens, and CD4^+^ cells were enriched using a CD4^+^ T mouse cell isolation kit (Miltenyi Biotec, Germany). Surface staining was performed with anti‐CD4 (GK1.5, Biolegend, USA) and anti‐CD25 (PC61, Biolegend, USA). CD4^+^CD25^+^Foxp3^+^ Treg were sorted using a FACS‐Aria III (BD Biosciences, USA) (Figure S1b).

### 5.2 Tissue‐Engineered Construct Preparation Prior to Implantation

Microporous biphasic calcium phosphate (BCP) granules (MBCP 0.5–1 mm; Biomatlante, France) were used as scaffolds. Forty milligrams of BCP granules were prewetted overnight in MSC culture medium to enhance nutrient absorption and cell attachment. MSC from passage 2 were seeded at 2 × 10^4^ cells/mg BCP (total 8 × 10^5^ MSC per construct) onto prewetted granules (BCP+MSC) and allowed to attach for 2–3 h. For Treg coculture, sorted CD4^+^CD25^+^Foxp3^+^ cells were preactivated for 1 h before coculture, by seeding at 3 × 10^6^/cm^2^ onto plates coated with anti‐CD3 (5 µg mL^−1^) and anti‐CD28 (2 µg mL^−1^) soluble monoclonal antibodies (R&D Systems, USA). Activation was performed in RMPI‐1640 (Gibco, Thermo Fischer Scientific, USA) supplemented with 10% FBS and incubated for 1 h at 5% CO_2_ incubator at 37°C. After activation, Treg were collected, centrifuged, counted, and added to MSC‐seeded constructs at a 1:1 MSC:Treg ratio (BCP+MSC+Treg) using standard MSC culture medium [72, 73]. Constructs were implanted after 36 h of coculture.

For RvE1 priming, MSC‐seeded constructs received 10 nm RvE1 (Cayman Chemicals, USA), based on previous studies [23, 74, 75]. Medium was replenished with fresh 10 nm RvE1 after 24 h. Constructs (BCP+MSC+RvE1) were implanted 36 h after initial MSC seeding. The experimental set up and groups are summarized in (Figure 1).

To preserve stability and bioactivity, all RvE1 handling followed established protocols for oxidation‐ and light‐sensitive lipid mediators. RvE1 was stored at −80°C in amber glass vials under nitrogen gas to minimize oxidation. Aliquots were prepared to avoid repeated freeze‐thaw cycles. Immediately before use, RvE1 was diluted from ethanol stock into sterile PBS in amber containers, kept on ice, and protected from light. Fresh dilutions were prepared daily.

### 5.3 Subcutaneous Implantation

Male and female Foxp3^DTR/eGFP^ mice (The Jackson Laboratory, USA; Strain#: 016958) (8–9 weeks old; *n* = 5–6 per group per time point) were anesthetized using Isoflurane (Isoba, UK). A 1‐cm midline dorsal incision was made, and two subcutaneous pockets were created, one on each side of the midline. Each pocket received a single construct; both constructs in each animal were of the same experimental group to ensure systemic immune responses reflected that group. Each mouse therefore received two implants of the same type. Constructs implanted included BCP, BCP+MSC, BCP+MSC+Treg, or BCP+MSC+RvE1. Group assignments were randomized, with allocation concealment. Skin incisions were sutured (Vicryl, Ethicon, USA), and mice were euthanized at 2 and 10 weeks. An untreated “baseline” group (no implants) was included to establish physiological steady‐state immune cell profiles.

These time points were selected to capture temporally distinct phases of immune modulation and bone regeneration at both gene and protein level. The early time point corresponds to the peak adaptive immune activation and early osteogenic matrix deposition [52]. While the later time point aligns with our previous findings, which demonstrate robust ectopic osteogenesis from 8 to 11 weeks postimplantation [76, 77], while reflecting the maturation and remodeling phase of bone formation.

### 5.4 Treg Depletion and RvE1 Local Delivery

To study the role of Treg in BCP+MSC osteogenesis, Foxp3^DTR/eGFP^ mice strain were used [78]. Foxp3^DTR/eGFP^ mice (8–9 weeks old) received diphtheria toxin (DT; Sigma‐Aldrich, USA) at 50 µg/kg injected intraperitoneally (i.p.) for two consecutive days postconstruct implantation, followed by injections on days 4, 6, 8, and 10. Treg depletion was confirmed in lymphoid organs (mesenteric, cervical, brachial lymph nodes, and spleen) by flow cytometry at day 0 and day 14 (Figure S1c). All animals (*n* = 12) received BCP+MSC constructs before depletion (at day 0) (group: (dep)BCP+MSC). Half (*n* = 6) received 1 µm RvE1 in 50 µL PBS, injected locally in the implantation site daily ((dep)BCP+MSC+RvE1inj). This dose was based on prior work demonstrating effective prevention of adaptive immune activation and bone loss with daily local 1 µm RvE1 administration [11]. Mice were euthanized 2 weeks after implantation.

### 5.5 Immunophenotyping by Flow Cytometry

Temporal immunophenotyping of major immune subsets in regional lymph nodes and spleen was conducted using multicolor flow cytometry to assess the local and systemic immune microenvironment response at early (2 weeks) and late (10 weeks) time points. Brachial lymph nodes (draining the implant site) and spleens were collected from mice. Single‐cell suspensions were obtained using 70 µm cell strainers (Sigma‐Aldrich, USA). For IFN‐γ detection, 4 × 10^6^ cells were stimulated for 4 h in RPMI‐1640 supplemented with 10% FBS, 1% penicillin‐streptomycin, 1 × Brefeldin A (eBioscience, USA), Phorbol 12‐myristate 13‐acetate (50 ng mL^−1^), and Ionomycin (1 µg mL^−1^) (Sigma‐Aldrich, USA). Cells were stained with LIVE/DEAD (Thermo Fisher Scientific, USA) for 30 min in the dark and Fc blocked using TruStain FcX antibody (Biolegend, USA) following the manufacturer's instructions. The following antibodies in PBS with 5% FBS were used for extracellular staining and incubated for 30 min at 4°C in the dark: anti‐CD11b, anti‐CD3ɛ, anti‐CD4, anti‐CD8a and anti‐CD19. Intracellular staining was performed using a fixation/permeabilization kit (eBioscience, USA) following the manufacturer's instructions, with anti‐IFN‐γ, anti‐CD4, and anti‐CD8a (all antibodies from Biolegend, USA) (Table S1 and Figure S1d). All fluorochromes in the panel were compensated in single stains using the AbC Total Antibody Compensation Bead Kit (Thermo Fisher Scientific, USA) according to the manufacturer's instructions. Forward scatter (FSC) and side scatter (SSC) Height and Area parameters were used. No issues were encountered with tandem dyes. Compensation was calculated during acquisition on an Attune NxT flow cytometer (Invitrogen, USA) and applied uniformly to all samples. Unstained controls were included to establish background autofluorescence and to assist in setting gates for positive populations. Unstimulated cells were included as controls to assess basal IFN‐γ expression. FMO controls were prepared for key markers but were not required due to clear separation between positive and negative populations and absence of rare or dim populations (Figure S1d).

### 5.6 High‐Dimensional Analysis of Flow Cytometry Data

Multiparametric data analysis was performed using FlowJo software v10.7.1 (CA, USA). T‐distributed stochastic neighbor embedding (*t*‐SNE) is a nonlinear dimensionality reduction algorithm able to visualize high‐dimensional data by giving each datapoint a location in a 2D or 3D map. The cells are visualized as points colored by clustering assignment. Samples were first manually pregated to exclude doublets, debris, and dead cells. This was followed by down sampling of the live cells to 10 000 events from each sample. Samples from all groups were then concatenated into the population of interest and dimensionally reduced in FlowJo (iterations 1000, perplexity 30, and learning rate (eta): 5). A *t*‐SNE representation was generated for each time point (2 and 10 weeks) with samples from all tested groups. Biological replicates: 2 weeks: *n* = 4; 10 weeks: *n* = 3–5; Treg‐depletion experiments: *n* = 5–6.

### 5.7 Bulk RNA Sequencing of Tissue‐Engineered Constructs

Scaffold constructs (*n* = 4 biological replicates) harvested at 2 weeks from all groups were processed for total RNA isolation using the RNeasy Mini Kit (Qiagen, Germany), following the manufacturer's instructions. cDNA libraries were prepared using the Truseq Stranded mRNA sample preparation kit. The libraries were sequenced on NovaSeq 6000 (paired end). Demultiplexing and data aggregation were performed using the Broad's Picard pipeline. The processed FASTQ files were mapped to the mouse genome using HISAT2 (version 2.0.5) [79]. The aligned bam files were used for reading quantification against annotations from gencode version M13 using the tool FeatureCounts (version 1.5.2) [80]. Differential expression analysis was carried out in DESeq2 version 1.42.0 package in R software (R Foundation for Statistical Computing, Austria) [81], which applies median‐of‐ratios normalization to correct for library‐size differences and RNA composition bias. This method calculates a size factor for each sample by taking the median ratio of observed counts to the geometric mean across all samples, ensuring comparable median expression levels between libraries. Normalized counts were then used for dispersion estimation, shrinkage of fold changes, and visualization figures. Significantly deregulated genes were defined by adjusted *p*‐value < 0.05 and a log2FC >1 for upregulated and log2FC < −1 for downregulated genes.

Pathway analysis for significantly up‐ and down‐regulated genes was performed by using R packages “clusterProlifer” [82] and “topGO” [83] to identify enrichment of gene ontology (GO) terms (including biological processes) and KEGG pathways.

### 5.8 Cytokine and Chemokine Profiling in Constructs and Serum

Protein was extracted from scaffold constructs at 2 and 10 weeks by shaking in RIPA buffer with 1 × Halt protease inhibitor cocktail and 1 × Halt phosphatase inhibitor cocktail (all from Thermo Fischer Scientific, USA) at 4°C for 20 min. After sonication and centrifugation at 16 000 g for 20 min at 4°C, protein content was quantified using BCA assay (Pierce BCA Protein assay kit, Thermo Fisher Scientific, USA), following the manufacturer's instructions. Cytokine/Chemokine levels were measured using a customized MILLIPLEX MAP Mouse Cytokine/Chemokine 19‐plex panel (Sigma‐Aldrich, USA) on a Bio‐Plex200 (Bio‐Rad, USA) following manufacturers’ specifications. Biological replicates: 2 weeks: *n* = 4; 10 weeks: *n* = 3.

### 5.9 Histological Analysis of Implanted Tissue‐Engineered Constructs

Scaffold constructs harvested at 2 and 10 weeks were fixed in 4% paraformaldehyde, decalcified in EDTA (Sigma‐Aldrich, USA), and paraffin‐embedded. Sections (3–4 µm) were stained with hematoxylin and eosin (H&E) (both from CellPath, UK) at RT using an automated stainer (SAKURA Tissue‐Tek Prisma Plus, Sakura Finetek, Japan) following a standard protocol. Briefly, slides were deparaffinized in Tissue Clear (Sakura Finetek, Japan) (10 min) after heat treatment (15 min), then rehydrated through graded ethanol and rinsed in water (1 min). Hematoxylin was applied for 5 min with agitation, followed by rinsing and eosin staining for 30 s. Slides were dehydrated through ethanol and cleared in xylene (Sigma‐Aldrich, USA) before cover slipping. To visualize and emphasize collagen and bone formation, Masson's Trichrome staining was utilized. Briefly, sections were deparaffinized in xylene and rehydrated through graded alcohol at room temperature. Slides were stained with Hematoxylin (2 min), rinsed in running water (5–10 min), stained with Ponceau (Sigma‐Aldrich, USA) (1 min) and rinsed in distilled water. Phosphotungstic acid hydrate (Sigma‐Aldrich, USA) was applied for 20 min, followed by rinse. Sections were then stained with 0.1% Fast Green (Sigma‐Aldrich, USA) for 1–2 min, rinsed, dehydrated through graded alcohols, cleared in xylene, and mounted. Slides were digitized using a NanoZoomer XR scanner (Hamamatsu, Japan) at 40× resolution, using a focus score function to evaluate image quality, not a traditional threshold algorithm generating NDPI files. Image quality was assessed using the scanner's automated focus scoring system, which evaluates clarity and flags slides for manual review or rescanning if necessary.

Qualitative histological evaluations were conducted to assess the host response and the ectopic bone formed. Fibrous capsule layers were quantified from six different regions per section. Biological replicates: 2 and 10 weeks: *n* = 5

### 5.10 Animal Ethical Statement

The animal studies were reviewed and approved by the Institutional Animal Care and Use Committee (IACUC) of The ADA Forsyth Institute (protocol #19.011). Constructs were randomly assigned to implantation sites, and the operator was blinded to group allocation. Both sexes (8–9 weeks old) were included without exclusions. All efforts were made to follow the 3Rs guidelines, minimizing animal use and suffering.

### 5.11 Statistical Analysis

Descriptive statistics were applied to screen for potential outliers. Continuous variables are reported as mean +/− standard error of the mean (SEM). Group differences were assessed using one‐way ANOVA, followed by Tukey and least significant difference (LSD) post hoc tests for multiple comparisons. Two‐group comparisons used independent *t*‐tests. Test of normality was performed for residuals after model fitting, using the Kolmogorov–Smirnov Test. Analyses were performed separately for each time point. In all analyses “n” refers to biological replicates (individual animals). Sample sizes reflect distribution across assays; exact numbers appear in corresponding figure legends, experimental and results sections. Analyses were conducted using SPSS version 22 (IBM, NY, USA) or GraphPad Prism v10 (GraphPad Software, San Diego, USA). A two‐sided *p*‐value of ≤ 0.05 was considered significant.

Data from male and female mice were pooled, as the study was designed to assess the effect of MSC priming on bone formation not sex‐specific differences. The sample size was based on prior comparable studies [26, 84, 85, 86] and was not powered to detect sex‐related effects.

## Funding

This work was funded by the Trond Mohn Foundation (Grant no. TMS2021STG03) and Research Council of Norway (Grant no. 314473). A.S is funded by Trond Mohn Foundation (Grant no. BFS2017TMT08).

## Conflicts of Interest

The authors declare no conflicts of interest.

## Supporting information




**Supporting File**: advs74654‐sup‐0001‐SuppMat.docx.

## Data Availability

All data are provided in figures, tables, supplementary information or are otherwise available upon request. All sequencing data generated in this study have been deposited in the Gene Expression Omnibus (GEO) and will be made publicly available upon publication under accession number GSE299815.
