# Supplementary material for: Distinct Immunomodulatory Strategies Guide Mesenchymal Stromal/Stem Cell‐Mediated Bone Regeneration
Source: Adv Sci (Weinh). 2026 Mar 5;13(27):e18026. doi: 10.1002/advs.202518026 (PMC13170215; doi:10.1002/advs.202518026)
Supplement: Supplementary file 1 — Supporting File: advs74654‐sup‐0001‐SuppMat.docx. [file ADVS-13-e18026-s001.docx]

Supporting Information

**Distinct Immunomodulatory Strategies Guide Mesenchymal Stromal/Stem Cell-Mediated Bone Regeneration**

*Salwa Suliman^1,2^*********, Carla Alvarez Rivas^2,3^, Aashish Srivastava^4^, Nora Marek^1^, Kamal Mustafa^1^, Alpdogan Kantarci^2,5^********

**Supplementary Figures**


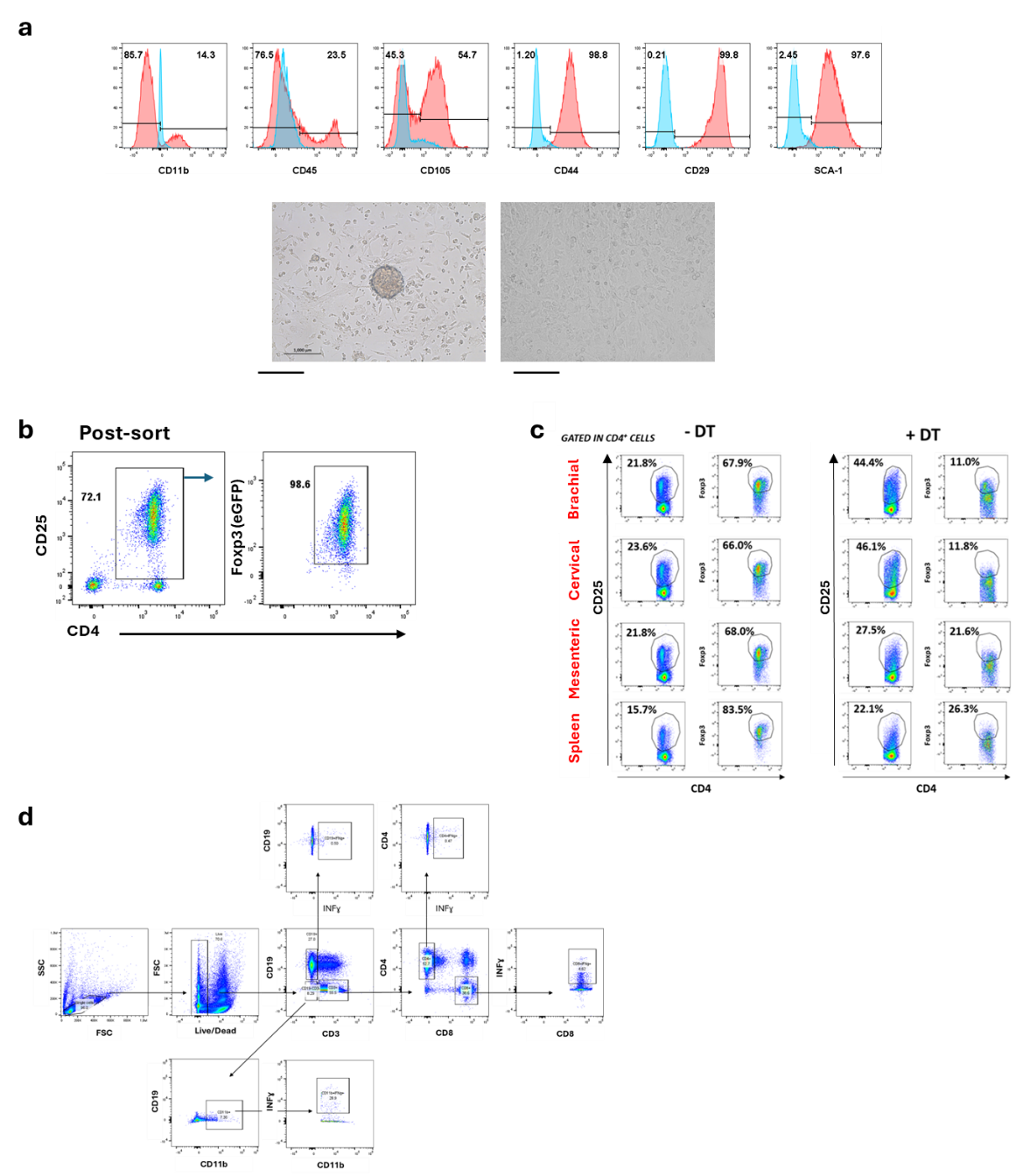


**Figure S1. (a)** Bone marrow-derived MSC phenotyping by flow cytometry showing CD11b and CD45 low, and CD105, CD44, CD29 and SCA-1 positive. Representative light microscopy images of MSC in culture. Scale bar = 1000 µm. **(b)** Post-sorting of Treg from Foxp3^DTR/eGFP^. **(c)** Confirmation of Treg depletion by flow cytometry, showing reduction of CD4^+^CD25^+^FOXP3^+^ cells in lymphoid organs. DT: diphtheria toxin. **(d)** Flow cytometry gating for identification of immune cell subsets.


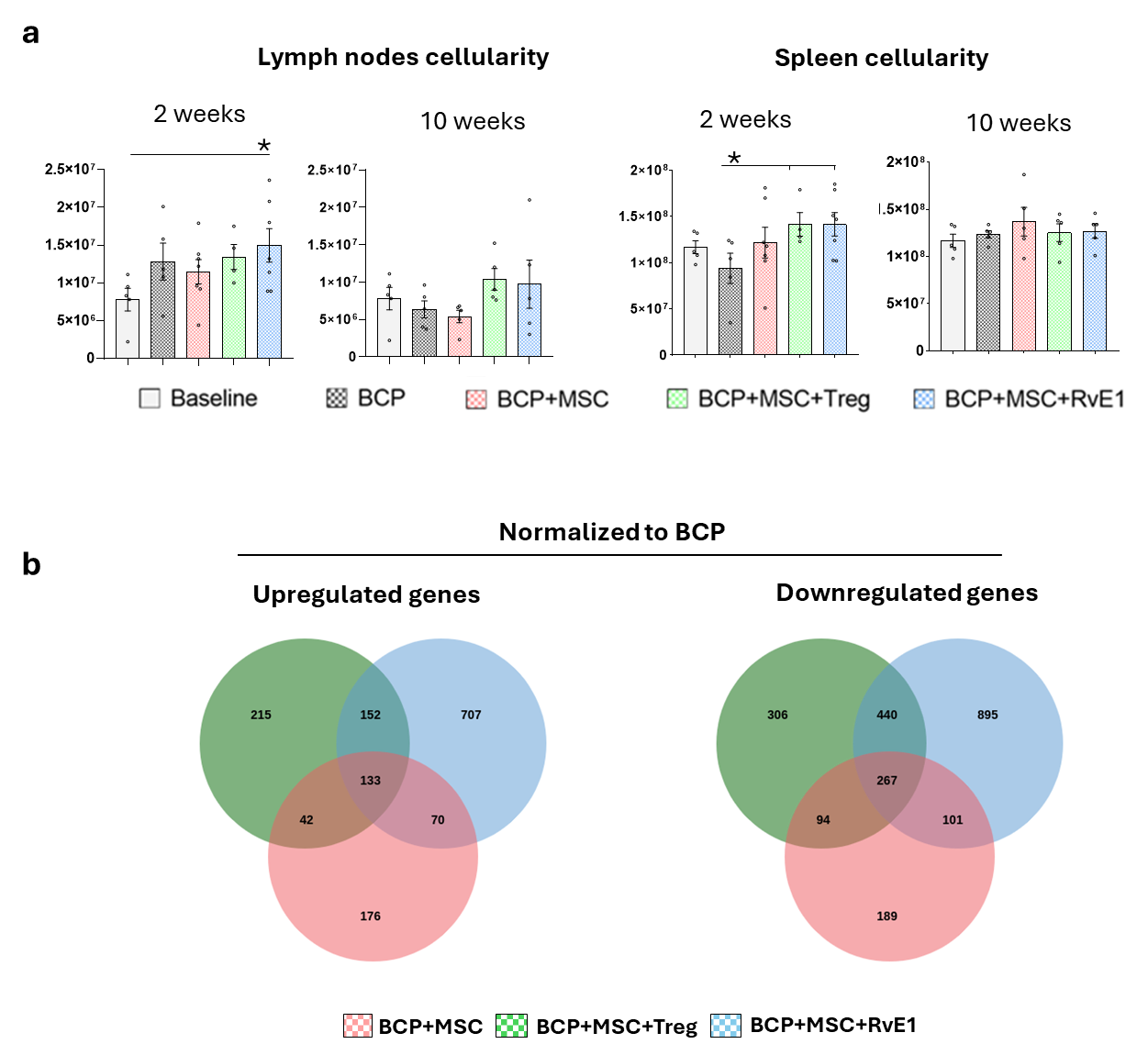


**Figure S2: (a)** Total cellularity in lymph nodes and spleen across experimental groups after 2 and 10 weeks. ‘Baseline’ refers to untreated, construct-free animals. Data are presented as mean +/- SEM (n = 4-6 animals per group). Statistical significance was determined by one-way ANOVA with post hoc tests. *p<0.05. **(b)** Venn diagrams showing upregulated and downregulated genes across experimental groups, normalized to the BCP group. N = 4 animals per group.


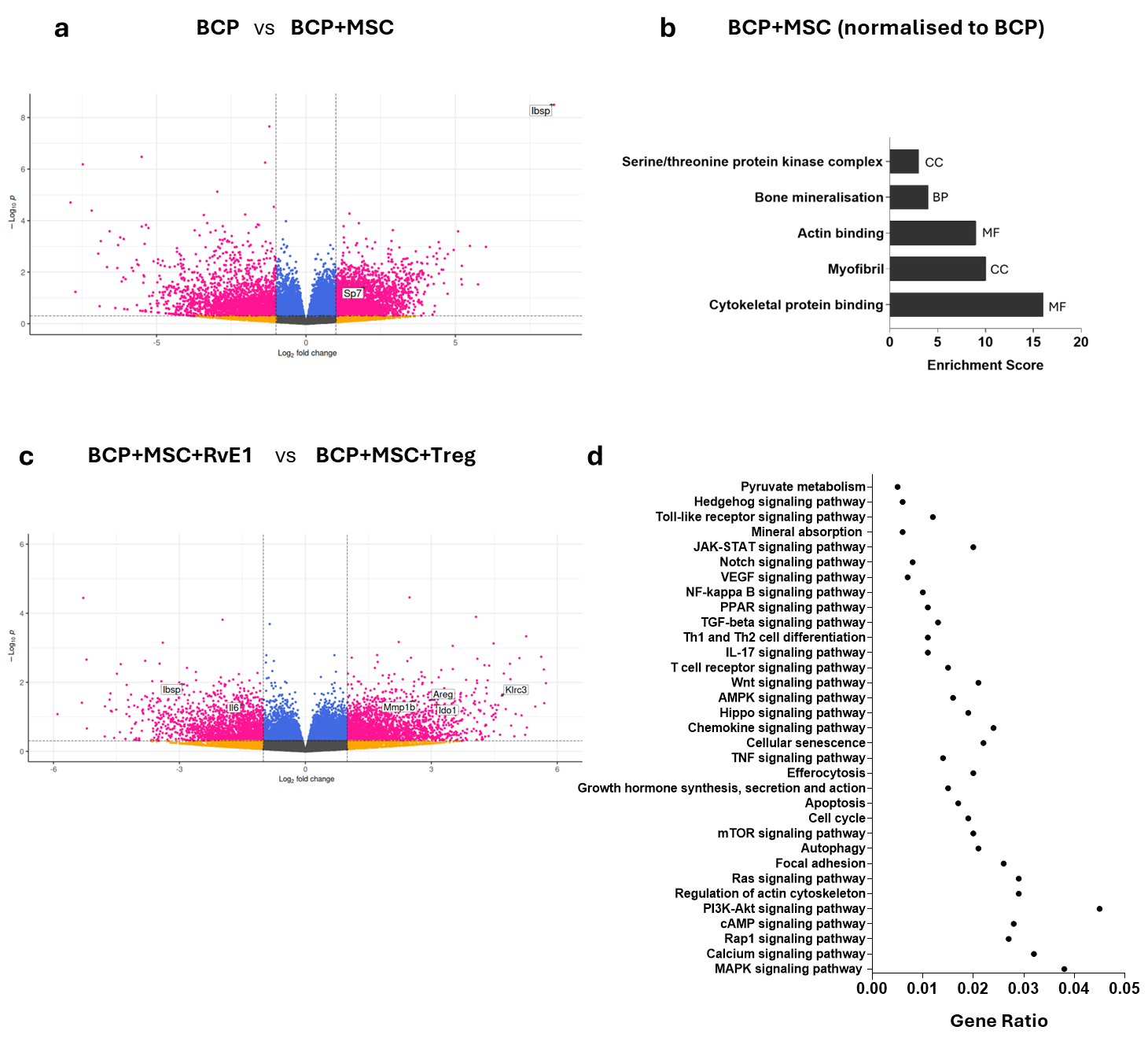


**Figure S3**. **(a)** Volcano plots depicting differential gene expression in BCP versus BCP+MSC, with -log_10_ on the y-axis and log_2_ fold change on the x-axis. **(b)** Enriched pathways in BCP+MSC normalized to BCP. **(c)** Volcano plots depicting differential gene expression in BCP+MSC+RvE1 versus BCP+MSC+Treg, with -log_10_ on the y-axis and log_2_ fold change on the x-axis. **(d)** Enriched pathways in BCP+MSC+Treg normalized to BCP+MSC+RvE1. N = 4 animals per group.


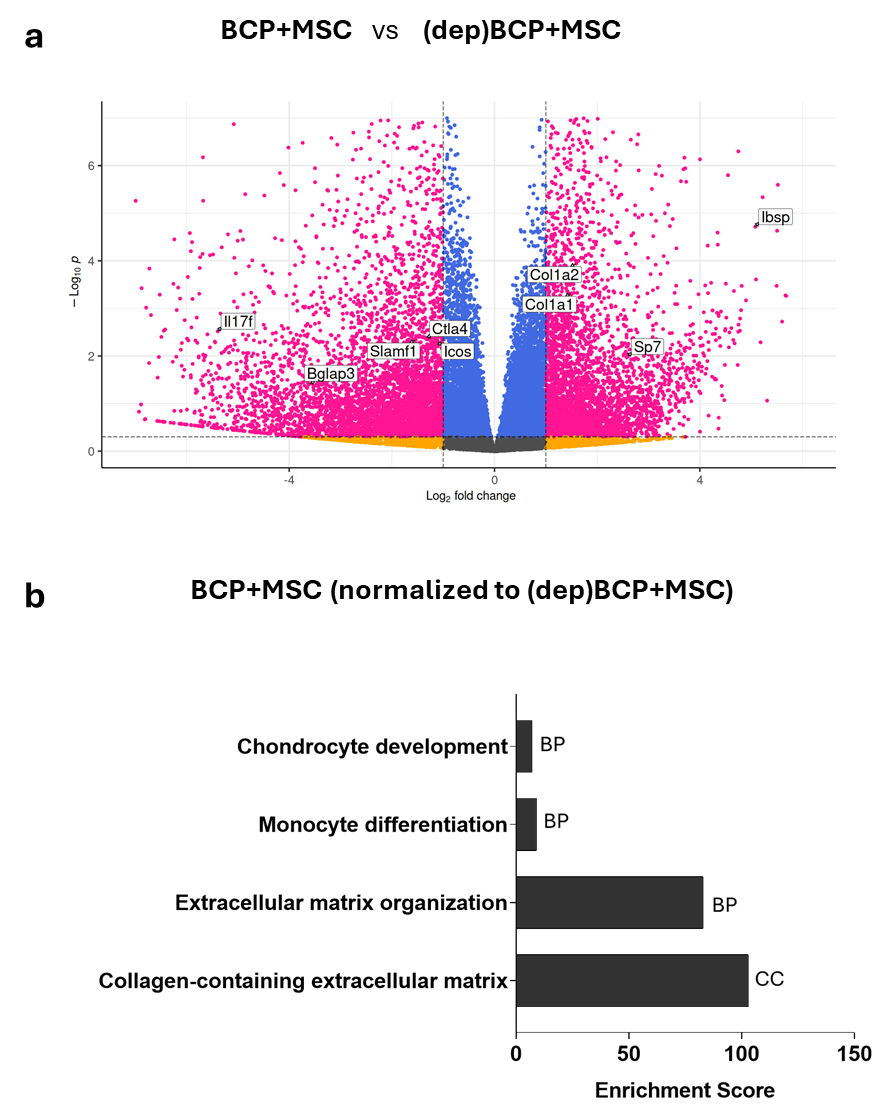


**Figure. S4. (a)** Volcano plots illustrating differential genes expression in BCP+MSC versus (dep)BCP+MSC, with -log_10_ on the y-axis and log_2_ fold change on the x-axis. **(b)** Enrichment pathways identifying functional modules in BCP+MSC normalized to (dep)BCP+MSC. BP: biological processes, CC: cellular component. N = 4 animals per group.


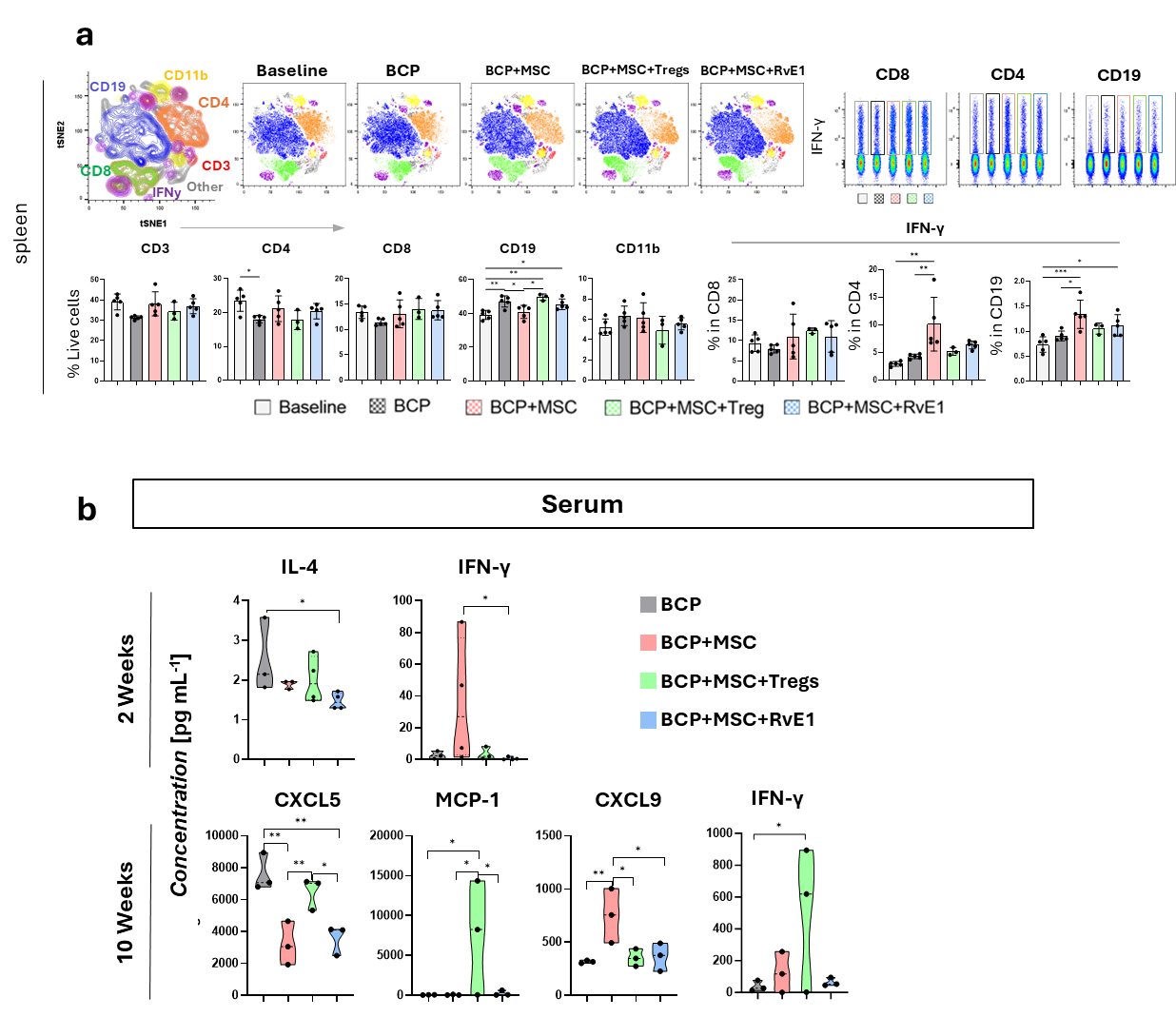


**Figure S5. (a)** Dimensional reduction using t-SNE of immune cells from spleen after 10 weeks, showing population distribution for each group. ‘Baseline’ refers to untreated, construct-free animals. Absolute quantification of each immune cell subset, along with absolute IFN-γ production, is shown. Data are presented as mean +/- SEM (n = 3-5 animals per group). Statistical significance was determined by one-way ANOVA with post hoc tests. *p<0.05, **p<0.01, ***p<0.001. **(b)** Quantification of significantly expressed cytokines and chemokines from serum after 2 and 10 weeks. Data are presented as means +/- SEM with n = 4 animals per group at 2 weeks and n = 3 animals per group at 10 weeks. Statistical significance was determined by one-way ANOVA with post hoc tests. *p<0.05, **p<0.01, ***p<0.001.

**Supplementary Tables**

**Table S1.** Antibodies used for flow cytometric staining.

| **Antibody** | **Clone** | **Company** | **Dilution** |
| --- | --- | --- | --- |
| Brilliant Violet 421™ anti-mouse CD19 | 6D5 | Biolegend | 1/200 |
| APC anti-mouse CD8a | 53-6.7 | Biolegend | 1/200 |
| APC/Cy7 anti-mouse CD3ε | 145-2C11 | Biolegend | 1/100 |
| Brilliant Violet 711™ anti-mouse CD4 | RM4-5 | Biolegend | 1/200 |
| PerCP/Cyanine5.5 anti-mouse/human CD11b | M1/70 | Biolegend | 1/200 |
| PE anti-mouse IFN-γ | XMG1.2 | Biolegend | 1/200 |
| CD105 PE/Cyanine7 | MJ7/18 | Biolegend | 1/50 |
| CD44 PE/Cyanine5 | IM7 | Biolegend | 1/200 |
| CD29 APC | HMβ1-1 | Biolegend | 1/200 |
| SCA-1 APC/Cyanine7 | D7 | Biolegend | 1/200 |
| PE anti-mouse CD45 | 30-F11 | Biolegend | 1/200 |

**Table S2.** Scaffold-associated cytokines and chemokines normalized to total protein content after 2 weeks *in vivo* implantation

| **Marker** |  | **Group** | **Average Level (pg µg^-1^)** | **SEM** | **P-value** |
| --- | --- | --- | --- | --- | --- |
| IL-1α | 1 | BCP | 0.112 | 0.056 |  |
|  | 2 | BCP+MSC | 0.088 | 0.0440 |  |
|  | 3 | BCP+MSC+Treg | 0.092 | 0.0458 |  |
|  | 4 | BCP+MSC+RvE1 | 0.122 | 0.061 |  |
|  | 5 | (dep)BCP+MSC | 0.135 | 0.068 |  |
|  | 6 | (dep)BCP+MSC+RvE1 | 0.106 | 0.053 |  |
| IL-2 | 1 | BCP | 0.109 | 0.054 |  |
|  | 2 | BCP+MSC | 0.106 | 0.053 |  |
|  | 3 | BCP+MSC+Treg | 0.119 | 0.060 |  |
|  | 4 | BCP+MSC+RvE1 | 0.119 | 0.060 |  |
|  | 5 | (dep)BCP+MSC | 0.117 | 0.059 |  |
|  | 6 | (dep)BCP+MSC+RvE1 | 0.113 | 0.057 |  |
| IL-6 | 1 | BCP | 0.097 | 0.049 | 1 – 3 p = 0.042 |
|  | 2 | BCP+MSC | 0.033 | 0.017 |  |
|  | 3 | BCP+MSC+Treg | 0.027 | 0.013 |  |
|  | 4 | BCP+MSC+RvE1 | 0.039 | 0.020 |  |
|  | 5 | (dep)BCP+MSC | 0.039 | 0.020 |  |
|  | 6 | (dep)BCP+MSC+RvE1 | 0.024 | 0.012 |  |
| IL-12 | 1 | BCP | 0.005 | 0.002 |  |
|  | 2 | BCP+MSC | 0.005 | 0.002 |  |
|  | 3 | BCP+MSC+Treg | 0.009 | 0.005 |  |
|  | 4 | BCP+MSC+RvE1 | 0.008 | 0.004 |  |
|  | 5 | (dep)BCP+MSC | 0.01 | 0.004 |  |
|  | 6 | (dep)BCP+MSC+RvE1 | 0.012 | 0.006 |  |
| IL-17 | 1 | BCP | 0.006 | 0.003 |  |
|  | 2 | BCP+MSC | 0.007 | 0.004 |  |
|  | 3 | BCP+MSC+Treg | 0.006 | 0.003 |  |
|  | 4 | BCP+MSC+RvE1 | 0.009 | 0.004 |  |
|  | 5 | (dep)BCP+MSC | 0.004 | 0.002 |  |
|  | 6 | (dep)BCP+MSC+RvE1 | 0.007 | 0.003 |  |
| IL-13 | 1 | BCP | 0.120 | 0.060 | 1 – 4 p = 0.016  2 – 4 p = 0.047 |
|  | 2 | BCP+MSC | 0.143 | 0.072 |  |
|  | 3 | BCP+MSC+Treg | 0.181 | 0.091 |  |
|  | 4 | BCP+MSC+RvE1 | 0.233 | 0.116 |  |
|  | 5 | (dep)BCP+MSC | 0.124 | 0.062 |  |
|  | 6 | (dep)BCP+MSC+RvE1 | 0.142 | 0.071 |  |
| IL-10 | 1 | BCP | 0.011 | 0.005 |  |
|  | 2 | BCP+MSC | 0.011 | 0.006 |  |
|  | 3 | BCP+MSC+Treg | 0.011 | 0.005 |  |
|  | 4 | BCP+MSC+RvE1 | 0.015 | 0.007 |  |
|  | 5 | (dep)BCP+MSC | 0.014 | 0.007 |  |
|  | 6 | (dep)BCP+MSC+RvE1 | 0.013 | 0.006 |  |
| IL-4 | 1 | BCP | 0.029 | 0.014 | 1 – 4 p = 0.005  2 – 4 p = 0.04 |
|  | 2 | BCP+MSC | 0.04659 | 0.023295 |  |
|  | 3 | BCP+MSC+Treg | 0.059 | 0.029 |  |
|  | 4 | BCP+MSC+RvE1 | 0.082 | 0.047 |  |
|  | 5 | (dep)BCP+MSC | 0.041 | 0.020 |  |
|  | 6 | (dep)BCP+MSC+RvE1 | 0.017 | 0.009 |  |
| CXCL10 | 1 | BCP | 0.104 | 0.052 |  |
|  | 2 | BCP+MSC | 0.126 | 0.0629 |  |
|  | 3 | BCP+MSC+Treg | 0.114 | 0.057 |  |
|  | 4 | BCP+MSC+RvE1 | 0.106 | 0.053 |  |
|  | 5 | (dep)BCP+MSC | 0.125 | 0.062 | 5 –6 p = 0.021 |
|  | 6 | (dep)BCP+MSC+RvE1 | 0.155 | 0.077 |  |
| CXCL1 | 1 | BCP | 0.165 | 0.082 | 1-2 p = 0.019  1- 4 p = 0.042 |
|  | 2 | BCP+MSC | 0.051 | 0.025 |  |
|  | 3 | BCP+MSC+Treg | 0.089 | 0.044 |  |
|  | 4 | BCP+MSC+RvE1 | 0.068 | 0.034 |  |
|  | 5 | (dep)BCP+MSC | 0.085 | 0.043 |  |
|  | 6 | (dep)BCP+MSC+RvE1 | 0.060 | 0.029 |  |
| CXCL5 | 1 | BCP | 1.589 | 0.794 | 1-2 p = 0.028  1-4 p = 0.025 |
|  | 2 | BCP+MSC | 3.779 | 1.890 |  |
|  | 3 | BCP+MSC+Treg | 2.931 | 1.466 |  |
|  | 4 | BCP+MSC+RvE1 | 3.841 | 1.920 |  |
|  | 5 | (dep)BCP+MSC | 1.883 | 0.941 |  |
|  | 6 | (dep)BCP+MSC+RvE1 | 3.060 | 1.530 |  |
| MCP-1 | 1 | BCP | 0.113 | 0.056 |  |
|  | 2 | BCP+MSC | 0.088 | 0.044 |  |
|  | 3 | BCP+MSC+Treg | 0.136 | 0.0678 |  |
|  | 4 | BCP+MSC+RvE1 | 0.077 | 0.038 |  |
|  | 5 | (dep)BCP+MSC | 0.356 | 0.178 | 5 – 6 p = 0.007 |
|  | 6 | (dep)BCP+MSC+RvE1 | 0.094 | 0.047 |  |
| CXCL9 | 1 | BCP | 0.602 | 0.301 |  |
|  | 2 | BCP+MSC | 0.586 | 0.292 |  |
|  | 3 | BCP+MSC+Treg | 0.812 | 0.406 |  |
|  | 4 | BCP+MSC+RvE1 | 0.753 | 0.376 |  |
|  | 5 | (dep)BCP+MSC | 0.655 | 0.327 | 5 – 6 p = 0.002 |
|  | 6 | (dep)BCP+MSC+RvE1 | 0.994 | 0.497 |  |
| CCL3 | 1 | BCP | 0.312 | 0.156 | 1 – 3 p = 0.028  1 – 4 p = 0.054 |
|  | 2 | BCP+MSC | 0.415 | 0.207 |  |
|  | 3 | BCP+MSC+Treg | 0.537 | 0.2687 |  |
|  | 4 | BCP+MSC+RvE1 | 0.5044 | 0.252 |  |
|  | 5 | (dep)BCP+MSC | 0.742 | 0.424 |  |
|  | 6 | (dep)BCP+MSC+RvE1 | 0.371 | 0.212 |  |
| CCL5 | 1 | BCP | 0.045 | 0.022 | 1- 3 p = 0.024  2- 3 p = 0.031  3- 4 p = 0.026 |
|  | 2 | BCP+MSC | 0.047 | 0.023 |  |
|  | 3 | BCP+MSC+Treg | 0.086 | 0.043 |  |
|  | 4 | BCP+MSC+RvE1 | 0.046 | 0.023 |  |
|  | 5 | (dep)BCP+MSC | 0.102 | 0.131 |  |
|  | 6 | (dep)BCP+MSC+RvE1 | 0.051 | 0.065 |  |
| GM-CSF | 1 | BCP | 0.011 | 0.005 |  |
|  | 2 | BCP+MSC | 0.007 | 0.003 |  |
|  | 3 | BCP+MSC+Treg | 0.009 | 0.004 |  |
|  | 4 | BCP+MSC+RvE1 | 0.009 | 0.004 |  |
|  | 5 | (dep)BCP+MSC | 0.009 | 0.004 |  |
|  | 6 | (dep)BCP+MSC+RvE1 | 0.009 | 0.004 |  |
| IFN-γ | 1 | BCP | 0.002 | 0.001 | 1-3 p = 0.03  2-3 p = 0.026  3-4 p = 0.050 |
|  | 2 | BCP+MSC | 0.002 | 0.001 |  |
|  | 3 | BCP+MSC+Treg | 0.007 | 0.003 |  |
|  | 4 | BCP+MSC+RvE1 | 0.003 | 0.001 |  |
|  | 5 | (dep)BCP+MSC | 0.148 | 0.074 | 5 – 6 p = 0.025 |
|  | 6 | (dep)BCP+MSC+RvE1 | 0.007 | 0.003 |  |
| TNF-α | 1 | BCP | 0.013 | 0.006 | 1- 4 p = 0.032 |
|  | 2 | BCP+MSC | 0.015 | 0.007 |  |
|  | 3 | BCP+MSC+Treg | 0.017 | 0.008 |  |
|  | 4 | BCP+MSC+RvE1 | 0.018 | 0.009 |  |
|  | 5 | (dep)BCP+MSC | 0.009 | 0.004 |  |
|  | 6 | (dep)BCP+MSC+RvE1 | 0.011 | 0.005 |  |
| VEGF | 1 | BCP | 0.009 | 0.0049 |  |
|  | 2 | BCP+MSC | 0.004 | 0.002 |  |
|  | 3 | BCP+MSC+Treg | 0.003 | 0.001 |  |
|  | 4 | BCP+MSC+RvE1 | 0.003 | 0.001 |  |
|  | 5 | (dep)BCP+MSC | 0.014 | 0.008 |  |
|  | 6 | (dep)BCP+MSC+RvE1 | 0.005 | 0.002 |  |
|  |  |  |  |  |  |

**Table S3.** Scaffold-associated cytokines and chemokines normalized to total protein content after 10 weeks *in vivo* implantation.

| **Marker** |  | **Group** | **Average Level (pg µg^-1^)** | **SEM** | **P-value** |
| --- | --- | --- | --- | --- | --- |
| IL-1α | 1 | BCP | 0.093 | 0.053 |  |
|  | 2 | BCP+MSC | 0.080 | 0.046 |  |
|  | 3 | BCP+MSC+Treg | 0.208 | 0.120 |  |
|  | 4 | BCP+MSC+RvE1 | 0.183 | 0.105 |  |
| IL-2 | 1 | BCP | 0.036 | 0.020 |  |
|  | 2 | BCP+MSC | 0.0621 | 0.035 |  |
|  | 3 | BCP+MSC+Treg | 0.037 | 0.021 |  |
|  | 4 | BCP+MSC+RvE1 | 0.043 | 0.025 |  |
| IL-6 | 1 | BCP | 0.031 | 0.017 |  |
|  | 2 | BCP+MSC | 0.012 | 0.007 |  |
|  | 3 | BCP+MSC+Treg | 0.055 | 0.031 |  |
|  | 4 | BCP+MSC+RvE1 | 0.033 | 0.019 |  |
| IL-12 | 1 | BCP | 0.004 | 0.002 | 1 – 3 p = 0.045 |
|  | 2 | BCP+MSC | 0.009 | 0.005 |  |
|  | 3 | BCP+MSC+Treg | 0.020 | 0.011 |  |
|  | 4 | BCP+MSC+RvE1 | 0.0125 | 0.007 |  |
| IL-17 | 1 | BCP | 0.004 | 0.002 |  |
|  | 2 | BCP+MSC | 0.002 | 0.001 |  |
|  | 3 | BCP+MSC+Treg | 0.0139 | 0.008 |  |
|  | 4 | BCP+MSC+RvE1 | 0.010 | 0.005 |  |
| IL-13 | 1 | BCP | 0.124 | 0.071 | 2-3 p = 0.013 |
|  | 2 | BCP+MSC | 0.080 | 0.046 |  |
|  | 3 | BCP+MSC+Treg | 0.199 | 0.114 |  |
|  | 4 | BCP+MSC+RvE1 | 0.133 | 0.077 |  |
| IL-10 | 1 | BCP | 0.009 | 0.005 |  |
|  | 2 | BCP+MSC | 0.009 | 0.005 |  |
|  | 3 | BCP+MSC+Treg | 0.029 | 0.016 |  |
|  | 4 | BCP+MSC+RvE1 | 0.021 | 0.012 |  |
| IL-4 | 1 | BCP | 0.006 | 0.003 |  |
|  | 2 | BCP+MSC | 0.009 | 0.005 |  |
|  | 3 | BCP+MSC+Treg | 0.011 | 0.006 |  |
|  | 4 | BCP+MSC+RvE1 | 0.003 | 0.002 |  |
| CXCL10 | 1 | BCP | 0.101 | 0.058 |  |
|  | 2 | BCP+MSC | 0.111 | 0.064 |  |
|  | 3 | BCP+MSC+Treg | 0.117 | 0.067 |  |
|  | 4 | BCP+MSC+RvE1 | 0.114 | 0.066 |  |
| CXCL1 | 1 | BCP | 0.072 | 0.042 |  |
|  | 2 | BCP+MSC | 0.043 | 0.025 |  |
|  | 3 | BCP+MSC+Treg | 0.071 | 0.041 |  |
|  | 4 | BCP+MSC+RvE1 | 0.047 | 0.027 |  |
| CXCL5 | 1 | BCP | 1.668 | 0.963 |  |
|  | 2 | BCP+MSC | 3.324 | 1.919 |  |
|  | 3 | BCP+MSC+Treg | 6.181 | 3.569 |  |
|  | 4 | BCP+MSC+RvE1 | 5.534 | 3.195 |  |
| MCP-1 | 1 | BCP | 0.075 | 0.043 |  |
|  | 2 | BCP+MSC | 0.056 | 0.0328 |  |
|  | 3 | BCP+MSC+Treg | 0.0678 | 0.0391 |  |
|  | 4 | BCP+MSC+RvE1 | 0.050 | 0.028 |  |
| CXCL9 | 1 | BCP | 0.857 | 0.494 |  |
|  | 2 | BCP+MSC | 1.036 | 0.598 |  |
|  | 3 | BCP+MSC+Treg | 1.499 | 0.866 |  |
|  | 4 | BCP+MSC+RvE1 | 1.053 | 0.608 |  |
| CCL3 | 1 | BCP | 0.307 | 0.177 |  |
|  | 2 | BCP+MSC | 0.209 | 0.121 |  |
|  | 3 | BCP+MSC+Treg | 0.297 | 0.171 |  |
|  | 4 | BCP+MSC+RvE1 | 0.164 | 0.095 |  |
| CCL5 | 1 | BCP | 0.032 | 0.018 |  |
|  | 2 | BCP+MSC | 0.037 | 0.021 |  |
|  | 3 | BCP+MSC+Treg | 0.067 | 0.039 |  |
|  | 4 | BCP+MSC+RvE1 | 0.045 | 0.026 |  |
| GM-CSF | 1 | BCP | 0.006 | 0.003 | 2 - 3 p = 0.044  2 - 4 p = 0.014 |
|  | 2 | BCP+MSC | 0.003 | 0.001 |  |
|  | 3 | BCP+MSC+Treg | 0.010 | 0.006 |  |
|  | 4 | BCP+MSC+RvE1 | 0.0128 | 0.007 |  |
| IFN-γ | 1 | BCP | 0.003 | 0.002 | \| 1-3 p = 0.024 \| \| --- \|   2-3 p = 0.024  3-4 p = 0.024 |
|  | 2 | BCP+MSC | 0.002 | 0.001 |  |
|  | 3 | BCP+MSC+Treg | 0.275 | 0.158 |  |
|  | 4 | BCP+MSC+RvE1 | 0.003 | 0.001 |  |
| TNF-α | 1 | BCP | 0.016 | 0.009 |  |
|  | 2 | BCP+MSC | 0.011 | 0.006 |  |
|  | 3 | BCP+MSC+Treg | 0.015 | 0.009 |  |
|  | 4 | BCP+MSC+RvE1 | 0.011 | 0.006 |  |
| VEGF | 1 | BCP | 0.003 | 0.002 |  |
|  | 2 | BCP+MSC | 0.004 | 0.0023 |  |
|  | 3 | BCP+MSC+Treg | 0.010 | 0.006 |  |
|  | 4 | BCP+MSC+RvE1 | 0.010 | 0.006 |  |
|  |  |  |  |  |  |

**Table S4**: Serum levels of detected cytokines and chemokines after 2 weeks *in vivo* implantation

| **Marker** |  | **Group** | **Average Level (pg ml^-1^)** | **SEM** | **P-value** |
| --- | --- | --- | --- | --- | --- |
| IL-1α | 1 | BCP | 1027.162 | 513.581 |  |
|  | 2 | BCP+MSC | 1094.3 | 547.15 |  |
|  | 3 | BCP+MSC+Treg | 862.537 | 431.268 |  |
|  | 4 | BCP+MSC+RvE1 | 765.542 | 382.771 |  |
|  | 5 | (dep)BCP+MSC | 593.486 | 342.649 |  |
|  | 6 | (dep)BCP+MSC+RvE1 | 452.237 | 226.118 |  |
| IL-6 | 1 | BCP | 4.75 | 2.742 |  |
|  | 2 | BCP+MSC | 11.662 | 5.831 |  |
|  | 3 | BCP+MSC+Treg | 3.415 | 1.707 |  |
|  | 4 | BCP+MSC+RvE1 | 3.727 | 1.863 |  |
|  | 5 | (dep)BCP+MSC | 214.73 | 123.974 |  |
|  | 6 | (dep)BCP+MSC+RvE1 | 28.235 | 14.117 |  |
| IL-17 | 1 | BCP | 7.3 | 3.65 |  |
|  | 2 | BCP+MSC | 6.012 | 3.006 |  |
|  | 3 | BCP+MSC+Treg | 7.402 | 3.701 |  |
|  | 4 | BCP+MSC+RvE1 | 4.127 | 2.063 |  |
|  | 5 | (dep)BCP+MSC | 5.772 | 2.886 |  |
|  | 6 | (dep)BCP+MSC+RvE1 | 11.335 | 5.667 |  |
| IL-13 | 1 | BCP | 109.18 | 63.035 |  |
|  | 2 | BCP+MSC | 111.986 | 64.655 |  |
|  | 3 | BCP+MSC+Treg | 139.59 | 80.592 |  |
|  | 4 | BCP+MSC+RvE1 | 72.527 | 36.263 |  |
|  | 5 | (dep)BCP+MSC | 368.8 | 598.007 |  |
|  | 6 | (dep)BCP+MSC+RvE1 | 184.4 | 299.003 |  |
| IL-4 | 1 | BCP | 2.516 | 1.452 | 1- 4 p = 0.03 |
|  | 2 | BCP+MSC | 1.896 | 1.095 |  |
|  | 3 | BCP+MSC+Treg | 2.007 | 1.003 |  |
|  | 4 | BCP+MSC+RvE1 | 1.475 | 0.737 |  |
|  | 5 | (dep)BCP+MSC | 6.575 | 3.287 |  |
|  | 6 | (dep)BCP+MSC+RvE1 | 8.767 | 4.383 |  |
| CXCL10 | 1 | BCP | 435.182 | 217.591 |  |
|  | 2 | BCP+MSC | 352.262 | 176.131 |  |
|  | 3 | BCP+MSC+Treg | 410.7 | 205.35 |  |
|  | 4 | BCP+MSC+RvE1 | 316.585 | 158.292 |  |
|  | 5 | (dep)BCP+MSC | 843.56 | 421.78 |  |
|  | 6 | (dep)BCP+MSC+RvE1 | 866.867 | 433.433 |  |
| CXCL1 | 1 | BCP | 154.64 | 77.32 |  |
|  | 2 | BCP+MSC | 201.285 | 100.642 |  |
|  | 3 | BCP+MSC+Treg | 172.022 | 86.011 |  |
|  | 4 | BCP+MSC+RvE1 | 136.755 | 68.377 |  |
|  | 5 | (dep)BCP+MSC | 194.407 | 87.59 |  |
|  | 6 | (dep)BCP+MSC+RvE1 | 97.203 | 43.795 |  |
| CXCL5 | 1 | BCP | 5236.405 | 2618.202 |  |
|  | 2 | BCP+MSC | 6970.83 | 3485.415 |  |
|  | 3 | BCP+MSC+Treg | 5656.24 | 2828.12 |  |
|  | 4 | BCP+MSC+RvE1 | 5643.897 | 2821.948 |  |
|  | 5 | (dep)BCP+MSC | 136.512 | 68.256 |  |
|  | 6 | (dep)BCP+MSC+RvE1 | 155.762 | 77.881 |  |
| MCP-1 | 1 | BCP | 54.797 | 27.398 |  |
|  | 2 | BCP+MSC | 76.405 | 38.202 |  |
|  | 3 | BCP+MSC+Treg | 42.012 | 21.006 |  |
|  | 4 | BCP+MSC+RvE1 | 30.252 | 15.126 |  |
|  | 5 | (dep)BCP+MSC | 136.512 | 155.762 |  |
|  | 6 | (dep)BCP+MSC+RvE1 | 68.256 | 77.881 |  |
| CXCL9 | 1 | BCP | 54.797 | 27.398 |  |
|  | 2 | BCP+MSC | 76.405 | 38.202 |  |
|  | 3 | BCP+MSC+Treg | 42.012 | 21.006 |  |
|  | 4 | BCP+MSC+RvE1 | 30.252 | 15.126 |  |
|  | 5 | (dep)BCP+MSC | 136.512 | 68.256 |  |
|  | 6 | (dep)BCP+MSC+RvE1 | 155.762 | 77.881 |  |
| CCL3 | 1 | BCP | 480.51 | 240.255 |  |
|  | 2 | BCP+MSC | 628.352 | 314.176 |  |
|  | 3 | BCP+MSC+Treg | 546.4 | 273.2 |  |
|  | 4 | BCP+MSC+RvE1 | 312.505 | 156.25 |  |
|  | 5 | (dep)BCP+MSC | 1809.327 | 904.663 | 5 -6 p = 0.012 |
|  | 6 | (dep)BCP+MSC+RvE1 | 2803.357 | 1401.678 |  |
| CCL5 | 1 | BCP | 376.342 | 188.171 |  |
|  | 2 | BCP+MSC | 40.685 | 20.342 |  |
|  | 3 | BCP+MSC+Treg | 35.415 | 17.707 |  |
|  | 4 | BCP+MSC+RvE1 | 15.09 | 7.545 |  |
|  | 5 | (dep)BCP+MSC | 60.625 | 30.312 |  |
|  | 6 | (dep)BCP+MSC+RvE1 | 48.59 | 24.295 |  |
| IFN-γ | 1 | BCP | 2.706 | 1.562 | 2 – 4 p = 0.047 |
|  | 2 | BCP+MSC | 35.58 | 17.79 |  |
|  | 3 | BCP+MSC+Treg | 3.603 | 2.080 |  |
|  | 4 | BCP+MSC+RvE1 | 0.6 | 0.3 |  |
|  | 5 | (dep)BCP+MSC | 76.262 | 38.131 |  |
|  | 6 | (dep)BCP+MSC+RvE1 | 74.447 | 37.223 |  |
| VEGF | 1 | BCP | 9.555 | 4.777 |  |
|  | 2 | BCP+MSC | 10.975 | 5.487 |  |
|  | 3 | BCP+MSC+Treg | 19.18 | 9.59 |  |
|  | 4 | BCP+MSC+RvE1 | 2.712 | 1.356 |  |
|  | 5 | (dep)BCP+MSC | 18.575 | 9.287 |  |
|  | 6 | (dep)BCP+MSC+RvE1 | 7.99 | 3.995 |  |
|  |  |  |  |  |  |

**Table S5.** Serum levels of detected cytokines and chemokines after 10 weeks *in vivo* implantation.

| **Marker** |  | **Group** | **Average Level (pg ml^-1^)** | **SEM** | **P-value** |
| --- | --- | --- | --- | --- | --- |
| IL-1α | 1 | BCP | 446.503 | 257.788 |  |
|  | 2 | BCP+MSC | 611.573 | 353.092 |  |
|  | 3 | BCP+MSC+Treg | 700.353 | 404.349 |  |
|  | 4 | BCP+MSC+RvE1 | 485.663 | 280.397 |  |
| IL-6 | 1 | BCP | 15.67 | 9.047 |  |
|  | 2 | BCP+MSC | 39.976 | 23.080 |  |
|  | 3 | BCP+MSC+Treg | 3443.51 | 1988.111 |  |
|  | 4 | BCP+MSC+RvE1 | 9.1 | 5.253 |  |
| IL-17 | 1 | BCP | 8.07 | 4.659 |  |
|  | 2 | BCP+MSC | 9.506 | 5.488 |  |
|  | 3 | BCP+MSC+Treg | 746.813 | 431.172 |  |
|  | 4 | BCP+MSC+RvE1 | 3.856 | 2.226 |  |
| IL-13 | 1 | BCP | 542.963 | 313.48 |  |
|  | 2 | BCP+MSC | 881.663 | 509.028 |  |
|  | 3 | BCP+MSC+Treg | 9602.893 | 5544.233 |  |
|  | 4 | BCP+MSC+RvE1 | 1340.166 | 773.745 |  |
| IL-4 | 1 | BCP | 5.8 | 3.348 |  |
|  | 2 | BCP+MSC | 41.693 | 24.071 |  |
|  | 3 | BCP+MSC+Treg | 1241.296 | 716.662 |  |
|  | 4 | BCP+MSC+RvE1 | 3.363 | 1.941 |  |
| CXCL10 | 1 | BCP | 364.36 | 210.363 |  |
|  | 2 | BCP+MSC | 351.556 | 202.971 |  |
|  | 3 | BCP+MSC+Treg | 418.763 | 241.773 |  |
|  | 4 | BCP+MSC+RvE1 | 429.59 | 248.023 |  |
| CXCL1 | 1 | BCP | 332.556 | 192.001 |  |
|  | 2 | BCP+MSC | 491.343 | 283.677 |  |
|  | 3 | BCP+MSC+Treg | 1319.746 | 761.956 |  |
|  | 4 | BCP+MSC+RvE1 | 349.823 | 201.970 |  |
| CXCL5 | 1 | BCP | 7610.06 | 4393.670 | 1 -2 p = 0.001  1 -4 p = 0.002  2 – 3 p = 0.007  3 – 4 p = 0.013 |
|  | 2 | BCP+MSC | 3207.37 | 1851.775 |  |
|  | 3 | BCP+MSC+Treg | 6496.9 | 3750.986 |  |
|  | 4 | BCP+MSC+RvE1 | 3574.736 | 2063.875 |  |
| MCP-1 | 1 | BCP | 63.44 | 36.627 | 2- 3 p = 0.034  1 – 3 p = 0.034  3 – 4 p = 0.038 |
|  | 2 | BCP+MSC | 65.586 | 37.866 |  |
|  | 3 | BCP+MSC+Treg | 7555.456 | 4362.144 |  |
|  | 4 | BCP+MSC+RvE1 | 262.863 | 151.764 |  |
| CXCL9 | 1 | BCP | 315.686 | 182.261 | 1 -2 p = 0.007  2 -3 p = 0.011  2 – 4 p = 0.013 |
|  | 2 | BCP+MSC | 751.69 | 433.988 |  |
|  | 3 | BCP+MSC+Treg | 353.253 | 203.950 |  |
|  | 4 | BCP+MSC+RvE1 | 364.426 | 210.401 |  |
| CCL3 | 1 | BCP | 77.197 | 44.569 |  |
|  | 2 | BCP+MSC | 208.873 | 120.593 |  |
|  | 3 | BCP+MSC+Treg | 1138.167 | 657.121 |  |
|  | 4 | BCP+MSC+RvE1 | 184.327 | 106.421 |  |
| CCL5 | 1 | BCP | 44.613 | 25.757 |  |
|  | 2 | BCP+MSC | 49.593 | 28.632 |  |
|  | 3 | BCP+MSC+Treg | 1891.313 | 1091.950 |  |
|  | 4 | BCP+MSC+RvE1 | 30.04 | 17.343 |  |
| IFN-γ | 1 | BCP | 38.5 | 22.227 | 1 – 3 p = 0.043 |
|  | 2 | BCP+MSC | 124.57 | 71.920 |  |
|  | 3 | BCP+MSC+Treg | 505.156 | 291.652 |  |
|  | 4 | BCP+MSC+RvE1 | 64.24 | 37.088 |  |
| VEGF | 1 | BCP | 25.163 | 14.528 |  |
|  | 2 | BCP+MSC | 47.92 | 27.666 |  |
|  | 3 | BCP+MSC+Treg | 220.966 | 127.575 |  |
|  | 4 | BCP+MSC+RvE1 | 30.7833 | 17.772 |  |
|  |  |  |  |  |  |
